# A Review of Genomic, Transcriptomic, and Proteomic Applications in Edible Fungi Biology: Current Status and Future Directions

**DOI:** 10.3390/jof11060422

**Published:** 2025-05-30

**Authors:** Muyun Xie, Jing Wang, Feixiang Wang, Jinfeng Wang, Yunjin Yan, Kun Feng, Baixiong Chen

**Affiliations:** School of Bioengineering, Zunyi Medical University, Jinwan Road No. 368, Zhuhai 519090, China; xiemujun@zmuzh.edu.cn (M.X.);

**Keywords:** genomics, transcriptomics, proteomics, edible fungi, metabolic networks, biosynthesis, molecular markers, stress resistance

## Abstract

Edible fungi, a group of globally significant macrofungi, are highly valued for their unique flavors and substantial nutritional and medicinal properties. Understanding the molecular mechanisms governing their growth, development, gene function, biosynthesis of valuable compounds, and environmental adaptation is crucial for enhancing yield and quality, providing essential scientific support for industrial progress. Genomics, transcriptomics, and proteomics, as cornerstone life science technologies, offer powerful, integrated approaches to decipher genetic codes, reveal gene expression patterns, and elucidate complex metabolic networks in edible fungi. These advancements are transitioning research from traditional cultivation methods towards deeper molecular biology exploration. This review synthesizes key progress in applying genomics, transcriptomics, and proteomics to edible fungi, with a particular focus on metabolism-related research and the fundamentals of metabolic network construction. It discusses how these technologies, independently and in preliminary integration, uncover critical steps and regulatory mechanisms within endogenous metabolic pathways. While acknowledging the importance of metabolomics and epigenomics as cutting-edge areas, this review focuses on the “classical triad” of genomics, transcriptomics, and proteomics due to their technological maturity, data accessibility, and established application base in elucidating core metabolic mechanisms in edible fungi. The goal is to deepen the understanding of edible fungi metabolic mechanisms, providing a vital theoretical basis and practical insights for optimizing cultivation, enabling genetic improvement, harnessing bioactive substances, and promoting industrial upgrading, thereby boosting the overall efficiency and competitiveness of the edible fungi industry.

## 1. Introduction

Edible fungi are generally defined as macroscopic edible fungi possessing distinct fruiting bodies or sclerotia and rich in nutrients and bioactive compounds. Globally, approximately 2300 species of edible fungi have been identified [1], primarily belonging to the phyla *Ascomycota* and *Basidiomycota*, with the latter constituting the majority [2]. Certain *Ascomycetes*, such as truffles (*Tuber* spp.) and *Cordyceps* species, are rich in active components like amino acids [3], nucleosides [4,5], polysaccharides [6], and sterols [7] and are recognized for various biological activities, including antioxidant [8], antitumor [9], antiviral, and antimicrobial effects [10]. The more commonly consumed *Basidiomycetes*, including shiitake (*Lentinula edodes*), button mushroom (*Agaricus bisporus*), and wood ear (*Auricularia* spp.), are generally abundant in carbohydrates, minerals, proteins, and diverse amino acids [11,12], exhibiting significant physiological activities, such as antioxidant [13], antitumor [14], hypoglycemic [15], and immune-enhancing properties. Consequently, edible fungi play an indispensable role in the human diet and health maintenance, fueling a thriving cultivation industry and making basic biological research on these organisms a prominent scientific focus.

**Figure 1:**
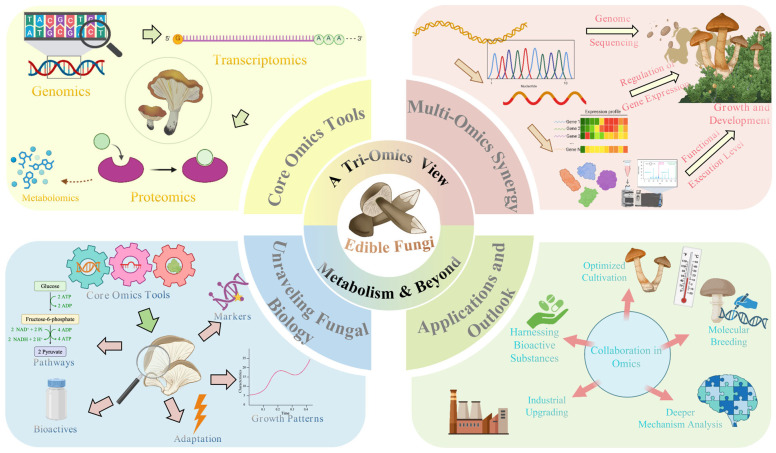
A framework illustrating genomic, transcriptomic, and proteomic applications in edible fungi: unraveling biology and metabolism, and guiding industrial advancement (created with BioGDP.com).

Against this backdrop, ‘Omics’ research has emerged, employing high-throughput platforms for the systematic acquisition and analysis of multi-dimensional biological data—including genomes, transcriptomes, proteomes, and metabolomes—from specific biological samples under defined conditions. The goal is to comprehend complex gene expression regulatory networks and metabolic systems holistically [16]. Initially achieving significant success in areas like disease etiology exploration [17], drug discovery [18], disease classification, and precision medicine/diagnostics [19], these high-throughput technologies have been increasingly integrated into edible fungi research. Studies now encompass a variety of important species, including *Agaricus bisporus*, *Auricularia* spp., *Flammulina velutipes*, *Ganoderma lucidum*, *Lentinula edodes*, *Pleurotus eryngii*, *Pleurotus ostreatus*, *Volvariella volvacea*, *Cordyceps militaris*, and *Morchella* spp. [20]. The application of multi-omics technologies is profoundly shifting edible fungi research from traditional reliance on morphological observation and cultivation experience towards fundamental investigations based on molecular evidence. This enables researchers to obtain complete DNA sequences, predict and identify key genes, and further investigate specific expression patterns, molecular genetic information, and functions using modern molecular biology tools like gene cloning, transgenesis, and gene editing [20]. These advancements are transitioning research from traditional cultivation methods towards deeper molecular biology exploration. While emerging technologies such as metabolomics (studying the global profile of small-molecule metabolites) and epigenomics, alongside comprehensive multi-omics integration, represent the cutting edge of systems biology research [16,20], the ‘classical triad’ of genomics, transcriptomics, and proteomics remains the cornerstone for elucidating life processes in edible fungi, particularly their core metabolic mechanisms (Figure 1). This is due to their technological maturity, relative data accessibility, and established application base. Genomics provides the genetic blueprint, transcriptomics reveals spatio-temporal gene expression dynamics [21], and proteomics directly reflects the status of functional effectors (proteins) and actual metabolic activity [22,23]. Information from these three levels is interconnected and hierarchical, providing indispensable insights into crucial questions: how edible fungi synthesize various nutrients, produce bioactive secondary metabolites, and respond to environmental changes through adaptive metabolic regulation. Such research, combined with genetic engineering, brings precision breeding for optimizing strain characteristics within reach. These advances not only deepen our understanding of gene function, molecular mechanisms, and regulatory networks at the micro-level but also enhance comprehension of macroscopic phenomena like phenotypic variation, growth, development, and pathological processes, ultimately serving molecularly informed breeding and scientific cultivation practices [24].

Genomics, transcriptomics, and proteomics each possess unique strengths and play pivotal roles in revealing gene function, regulating growth and development, managing stress responses, analyzing protein interaction networks, and elucidating biosynthetic pathways of active substances in edible fungi [25,26,27,28]. This review aims to summarize the current applications of genomics, transcriptomics, and proteomics in the basic research of common edible fungi (Figure 1). We will focus particularly on how these technologies, applied both individually and in initial combinations, are utilized to construct and analyze metabolic networks. We explore their contributions to dissecting key metabolic pathways, enzyme functions, and regulatory mechanisms. Through this review, we hope to provide valuable references and guidance for research focused on the genetic improvement of edible fungi, enhancement of bioactive compound yields, optimization of cultivation practices, and improvement of stress resistance, with the ultimate goal of facilitating quality enhancement and upgrading the entire edible fungi industry.

## 2. Genomics of Edible Fungi: Decoding the Genetic Basis of Metabolic Potential

The genome encompasses the entire genetic information of an organism, serving as the fundamental blueprint for its life activities. For edible fungi, resolving their genome sequences not only allows for the identification of species-specific characteristic sequences to support accurate taxonomic studies [29,30] but, more importantly, enables the mining of genes playing crucial roles in growth, development, environmental adaptation, and complex metabolic networks [31]. The composition and expression of these genes directly determine the biological characteristics of edible fungi, including growth rate, morphogenesis, stress tolerance, and the highly valued capacity for secondary metabolite biosynthesis [32]. Therefore, in-depth genomic research on edible fungi constitutes the logical starting point for understanding their metabolic potential and regulatory mechanisms [20]. With the advancement of gene editing technologies, targeted modification of key functional genes based on genomic information has become a significant pathway for the artificial selection of superior strains, enhancing their nutritional value and environmental adaptability [33] (Table 1).

### 2.1. Advances in Genome Sequencing Technology and Information Mining

The maturation and declining cost of High-Throughput Sequencing (HTS) technologies have profoundly propelled genomic research in edible fungi. Whole Genome Sequencing (WGS) has laid a solid foundation for deciphering the complete genetic codes of numerous edible fungi species [20]. In recent years, the genomes of various important edible fungi have been sequenced, including *Flammulina velutipes* [60], *Ganoderma lucidum* [61], *Sparassis latifolia* [54], *Morchella* spp. [50], and *Pleurotus giganteus* [53]. Furthermore, the mitochondrial genome sequences of species such as *G. lucidum* [62], *Pleurotus eryngii* [63], *Pleurotus ostreatus* [64], *Volvariella volvacea* [65], *Lentinula edodes* [29], *Russula griseocarnosa* [66], *Coprinellus micaceus* [67], and *Grifola frondosa* [68] have also been determined. Obtaining high-quality, high-completeness genome sequences is a prerequisite for subsequent precise gene function annotation, comparative genomics analysis, and metabolic pathway reconstruction. For instance, by continuously optimizing sequencing strategies (e.g., combining second- and third-generation sequencing) and assembly algorithms, researchers have obtained more accurate and contiguous genome sequence maps for *F. velutipes* [69,70] and *V. volvacea* [71]. A high-quality WGS project for *Lyophyllum decastes*, an edible and medicinal fungus widely cultivated in China, not only predicted approximately 14,499 gene models but also specifically identified a substantial repository of Carbohydrate-Active enZyme (CAZyme) genes, particularly within the laccase gene family [48]. Similarly, sequencing the genome of *Dictyophora indusiata* revealed a vast gene repertoire (about 19,909 genes), including 369 cytochrome P450 (CYP450) family genes and as many as 64 secondary metabolism gene clusters associated with terpenoid synthesis, directly implying significant potential for producing medicinally valuable compounds [40]. The genome sequencing of *Oudemansiella raphanipes* identified about 21,308 protein-coding genes, with 56 predicted to participate in the biosynthesis of important secondary metabolites (e.g., terpenes, polyketides, non-ribosomal peptides, siderophores). Its genome also harbors abundant CAZyme genes, indicating strong lignocellulose degradation capabilities and potential for producing bioactive substances [52]. These examples clearly demonstrate that identifying gene families involved in substance degradation (e.g., Carbohydrate-active enzymes, CAZymes) and synthesis (e.g., secondary metabolite gene clusters) is a common focus in current edible fungi genomics research, highlighting the importance of these genes for understanding fungal ecological functions and tapping into their application potential.

Building upon reference genome sequences, Whole Genome Resequencing (WGR) has emerged as a critical tool for uncovering intra-specific genetic variation, understanding population genetic structure, and mining trait-associated genes [72]. By comparing differences (e.g., SNPs, InDels, SVs) between individuals or populations and the reference genome, WGR provides an effective means to dissect the genetic basis of variations in metabolic traits. For example, resequencing studies on albino mutants of *C. militaris* successfully localized several mutated genes associated with aberrant pigment metabolism (e.g., the CmPKS gene) and found that specific mutations affected light-induced melanin accumulation, offering new insights into the molecular mechanism of albinism in this species [73]. Another study utilized WGR to analyze heat-tolerant mutants of *L. edodes* generated via *Agrobacterium tumefaciens*-mediated transformation (ATMT). It was discovered that T-DNA insertion sites were sometimes located in non-coding regulatory regions, suggesting that variations in non-coding regions can also significantly impact important traits (like heat tolerance, potentially related to the thermostability of stress metabolism-associated enzymes) and underscoring the importance of searching for key regulatory elements within these regions [74]. Due to a longstanding lack of effective genetic and molecular tools for *Hericium erinaceus*, its strain improvement has progressed relatively slowly. By resequencing an F1 single-spore isolation population, researchers successfully constructed a high-resolution genetic map for *H. erinaceus*. This laid a solid foundation for subsequent localization and cloning of Quantitative Trait Loci (QTLs) associated with the synthesis of important medicinal compounds (e.g., hericenones, erinacines), strongly supporting molecular marker-assisted breeding in this species [75]. Furthermore, a population genomics resequencing study involving wild and commercially cultivated *L. edodes* from China and other parts of the world not only revealed the primary genetic origins of cultivated Chinese shiitake but also discovered unique gene pools within wild populations from Southwest and Northeast China. This provides valuable information for the conservation of *L. edodes* germplasm resources and the exploration of desirable genes (e.g., those related to flavor compound synthesis or environmental adaptation metabolism) [76]. Resequencing applications have also extended into genetic engineering. For instance, by combining different transformation and screening techniques, alongside obtaining more precise genome information for *F. velutipes* [49,50] and *V. volvacea* [71], researchers successfully tested and mapped insertion sites for multiple exogenous genes in *F. velutipes* transformants [77]. This is significant for understanding insertion site preferences, optimizing transformation systems, and ensuring transgenic safety. Collectively, these research outcomes underscore a paradigm shift: genome (re)sequencing transcends mere data acquisition. Its true analytical power lies in the deep mining of embedded information concerning genetic diversity, functional genes, regulatory elements, and evolutionary history. The broader implication is a more nuanced understanding of how genomic landscapes shape metabolic capacities and adaptive traits, paving the way for knowledge-based strain improvement and resource exploitation.

As edible fungi research continues to deepen and sequencing technologies undergo continuous innovation, genome and resequencing analyses will persistently generate vast amounts of data. Future research will increasingly focus on refining genome sequence maps. This includes integrating technologies like optical mapping and High-throughput Chromosome Conformation Capture (Hi-C) to construct chromosome-level physical maps, anchoring these to genetic maps and ultimately achieving the integration of genome sequence maps, physical maps, and high-density linkage maps (‘three maps into one’). This will dramatically improve the precision of gene localization. Concurrently, utilizing high-quality reference genomes to thoroughly analyze developmentally abnormal mutants or compare the genomic variation patterns of specific strains under different environmental pressures (e.g., temperature, humidity, disease) will aid in decoding the formation mechanisms of complex traits. This will provide more precise guidance for the targeted improvement of desired traits and related breeding efforts. Furthermore, a persistent challenge impacting the utility of genomic data is the completeness of functional annotation. Even with advancements, the genomes of many edible fungi contain a significant number of genes with unknown functions, often labeled as “hypothetical proteins”. This directly limits the downstream functional interpretation derived from genomic blueprints and complicates the integration with transcriptomic and proteomic data, especially for non-model edible fungi species. Ongoing efforts in genome sequencing and annotation projects are continually working to address this gap.

### 2.2. Functional Annotation of Edible Fungi Genomes

Following genome sequencing, the subsequent critical step is functional annotation: identifying various functional elements within the genome (such as protein-coding genes, non-coding RNA genes, regulatory sequences, etc.) and predicting their biological functions. Functional genomics, built upon the information provided by structural genomics, is the primary research field dedicated to interpreting genetic information and elucidating gene functions at a holistic level in the post-genomic era [78,79]. A core objective of functional annotation in edible fungi research is the identification of genes encoding key enzymes, transporters, and regulatory factors within metabolic networks, laying the groundwork for subsequent metabolic pathway analysis and functional validation. Currently, functional annotation primarily relies on bioinformatics approaches, predicting potential gene functions by comparing predicted sequences against major public databases (e.g., NCBI NR, Swiss-Prot, GO, KEGG, Pfam). However, limitations in functional annotation persist, especially for non-model edible fungi, where the genomes may contain numerous genes of unknown function or “hypothetical proteins”, directly constraining the functional interpretation and integration of subsequent transcriptomic and proteomic data. Ongoing sequencing and annotation projects, such as those available through databases like MycoCosm (JGI) and FungiDB (VEuPathDB), are crucial for improving this aspect.

Throughout their life cycle, edible fungi synthesize and degrade various substances, a process reliant on a vast array of functionally diverse enzymes. Enzymes, acting as highly efficient biological catalysts under mild conditions with high substrate specificity, are deeply involved in nearly all life processes, including metabolism, nutrient acquisition, energy conversion, and signal transduction. Beyond the core metabolic enzymes essential for sustaining life, many edible fungi, particularly wood-rot species, synthesize numerous enzymes associated with the degradation of major plant cell wall components (lignocellulose). Enzymes such as cellulases, hemicellulases, and lignin-degrading enzymes not only assist fungi in obtaining carbon and energy from their environment but also possess significant industrial potential for applications in bioenergy, environmental protection, food processing, textiles, and paper manufacturing [80]. Consequently, the identification and annotation of various enzyme gene families within edible fungi genomes are paramount in functional genomics research.

#### 2.2.1. Identification of Carbohydrate-Active enZymes (CAZymes)

The Carbohydrate-Active enZYmes (CAZymes) database is the authoritative resource for annotating enzymes involved in carbohydrate metabolism. CAZymes are a broad class of enzymes involved in the synthesis, modification, and breakdown of carbohydrates. In virtually all sequenced edible fungi genomes, extensive and diverse repertoires of CAZyme genes have been discovered. These genes encode enzymes primarily belonging to Glycoside Hydrolases (GHs), GlycosylTransferases (GTs), Polysaccharide Lyases (PLs), Carbohydrate Esterases (CEs), and enzymes with Auxiliary Activities (AAs, such as lignin-degrading oxidases) [45,81]. For instance, analysis of the *Hericium erinaceus* genome identified 341 candidate CAZyme genes, comprehensively covering the major enzyme families required for degrading cellulose, hemicellulose, and lignin [45]. Genomes of various other edible fungi, including *Flammulina velutipes* [41], *Lentinula edodes* [47], *Volvariella volvacea* [81], *Termitomyces albuminosus* [82], as well as the previously mentioned *Lyophyllum decastes* [48], *Oudemansiella raphanipes* [52], and *Stropharia rugosoannulata* [55], have been successfully annotated to contain numerous enzyme genes involved in lignocellulose or hemicellulose degradation, such as cellulases, xylanases, mannanases, laccases, and manganese peroxidases [34,41,47,81,82]. The composition and abundance of these enzymes directly dictate the fungus’ ability to decompose and utilize substrates (especially plant fiber-rich materials), forming the basis of carbon and energy acquisition for primary metabolism. Concurrently, genome annotation also reveals members of GT families, which are involved not only in synthesizing oligosaccharides and glycoconjugates but also in the biosynthesis of the fungus’ own cell wall polysaccharides (e.g., β-glucans, chitin) [83], crucial for maintaining cell structure and morphology.

#### 2.2.2. Identification of Secondary Metabolism-Related Enzymes

Edible fungi are renowned for producing structurally diverse secondary metabolites with various bioactivities, often representing the source of their medicinal value. Genome annotation is the first step toward exploring this biosynthetic potential and identifying key enzyme-encoding genes. Major enzyme families involved in secondary metabolism include Cytochrome P450 monooxygenases (CYP450s, involved in diverse modification reactions), terpene synthases/cyclases (synthesizing terpene skeletons), Polyketide Synthases (PKSs, synthesizing polyketides), Non-Ribosomal Peptide Synthetases (NRPSs, synthesizing peptides), and various transferases (e.g., methyl-, acetyl-, glycosyltransferases, involved in structural modifications). For example, the significant expansion of the P450 gene family and the identification of multiple putative terpene synthase genes in the *Ganoderma lucidum* genome are thought to be closely related to its ability to synthesize an exceptionally rich array of ganoderic acids (a class of triterpenoids) [38,72]. Similarly, numerous P450 genes and terpene synthesis-related gene clusters were found in the *Dictyophora indusiata* genome [40]. Annotation of the *O. raphanipes* genome also predicted genes involved in the biosynthesis of terpenes, polyketides, NRPSs, and siderophores (a specific type of secondary metabolite) [52]. Phylogenetic analysis of the *Cordyceps militaris* genome identified not only 61 protease families but also genes involved in terpene cyclases, terpene synthases, fatty acid synthases, and geranylgeranyl diphosphate synthase, all potentially related to its synthesis of adenosine, cordycepin, and possibly terpenoids [38]. Key enzyme genes associated with L-lysine synthesis, involving potential diaminopimelate (DAP) and aminoadipate (AA) pathways, were annotated in the *F. velutipes* genome [59]. Functional annotation of *Agaricus blazei* indicated relatively active glycolysis, gluconeogenesis, and glyoxylate metabolism during the mycelial stage, whereas the fruiting body stage showed higher activity related to stress response, ribosome biogenesis, arginine synthesis, and steroid biosynthesis potentially linked to β-1,3-glucan synthesis [83]. Genome analysis of *Sparassis latifolia* also revealed, besides numerous carbohydrate metabolism enzyme genes, the presence of genes participating in the synthesis pathways of indole, terpene, and type I polyketide secondary metabolites [54]. Mining and annotating enzyme genes in edible fungi genomes, particularly CAZymes and secondary metabolism enzymes, not only facilitate a comprehensive understanding of their fundamental decomposition, synthesis, and metabolic capabilities at the micro-level but also provide guidance for subsequent in-depth investigations into gene regulatory networks, growth characteristics, and biosynthetic pathways of key active compounds. This information establishes a solid theoretical foundation for screening critical genes, performing functional validations, applying gene editing technologies, and ultimately serving the molecular breeding and efficient cultivation of edible fungi.

#### 2.2.3. Identification of Metabolic Regulatory Genes

In addition to enzyme genes directly participating in metabolic reactions, genome annotation can identify numerous potential regulatory genes. These genes finely tune the direction and rate of metabolic flux by controlling the timing and intensity of structural gene expression. Key regulatory genes include various Transcription Factors (TFs) [84], such as members of the widely distributed Zn(II)2Cys6 zinc cluster protein family in fungi [85], as well as crucial components of signal transduction pathways, like G protein-coupled receptors (GPCRs) [86], G protein subunits [87], Mitogen-Activated Protein Kinase (MAPK) pathway components [88], and calcium signaling pathway-associated proteins [89]. Identifying these regulatory genes is crucial for understanding the overall control logic of metabolic networks.

#### 2.2.4. Functional Annotation of the Mitochondrial Genome

The mitochondrion, the cell’s “powerhouse”, possesses its own relatively small genome that encodes essential proteins for energy metabolism (aerobic respiration). Functional annotation of the mitochondrial genome (mitogenome) primarily focuses on genes encoding subunits of the respiratory chain complexes (Complexes I to IV, e.g., NADH dehydrogenase subunits, cytochrome b, cytochrome c oxidase subunits) and ATP synthase (Complex V) subunits [41,43,48,81]. Furthermore, the mitogenome encodes rRNAs and some tRNAs required for mitochondrial ribosomes [63,68]. Resolving the coding content of the mitogenome helps elucidate its roles in cellular energy supply, maintaining mitochondrial replication, and potential involvement in processes like fungal drug resistance and cellular senescence [82,83]. For instance, sequencing and annotation of mitogenomes from species like *Pleurotus cornucopiae* [90], *P. eryngii* [63], *Grifola frondosa* [68], and *Sparassis* spp. [91] have identified these conserved protein-coding, rRNA, and tRNA genes. Although the amount of genetic information encoded by the mitogenome is far less than the nuclear genome, it still exerts significant regulatory influence on the growth, development, energy metabolism, and environmental adaptation of edible fungi. Exploring its functional genes contributes to a more comprehensive understanding of edible fungi biology.

In summary, genome functional annotation provides a “blueprint” or “parts list” of the metabolic capabilities of edible fungi. It systematically reveals their potential enzymatic capacities, secondary metabolite synthesis potential, and possible regulatory mechanisms. This serves as an indispensable foundation for subsequent transcriptomic, proteomic, and metabolomic studies, as well as for metabolic pathway reconstruction and experimental functional validation.

### 2.3. Influence of Edible Fungi Genomic Features and Comparative Genomics on Metabolic Network Construction

The structural characteristics of edible fungi genomes—such as gene arrangement, gene family size, and the presence of gene fusions or gene clusters—exert a profound influence on the constitution and evolutionary trajectory of their metabolic networks, ultimately shaping their unique metabolic product profiles. Comparative genomics, through the analysis of genomes from different species or distinct strains of the same species, reveals both conserved and specific elements arising during genome evolution. This approach elucidates how the expansion and contraction of gene families have molded the metabolic adaptability of species and identifies genetic variations linked to specific metabolic phenotypes.

#### 2.3.1. Gene Fusion and Metabolic Pathway Evolution

Gene fusion is a significant evolutionary event that can generate proteins with novel functions or optimize existing metabolic pathways. During investigations into the biosynthesis of *Ganoderma lucidum* triterpenes (ganoderic acids), researchers identified a unique gene formed by the fusion of the gene encoding lanosterol synthase (catalyzing a key step in triterpene synthesis) with a gene encoding a membrane protein [42]. This fusion event, likely occurring during the evolution of the *Ganoderma* genus, is hypothesized to have had a significant impact on the efficiency or diversity of triterpene synthesis, representing a distinct mechanism of metabolic pathway evolution.

#### 2.3.2. Gene Clusters (SMGCs) and Coordinated Regulation of Secondary Metabolite Synthesis

In fungi, including edible species, genes involved in the biosynthesis of specific secondary metabolites—comprising synthetic enzymes, regulatory genes, and transporter genes—are often physically clustered together on the genome, forming biosynthetic gene clusters (BGCs), also referred to as Secondary Metabolite Gene Clusters (SMGCs). This physical linkage is thought to facilitate the co-expression and coordinated regulation of these functionally related genes, thereby enabling the efficient and precise synthesis of specific metabolites. For example, the genome of *Cordyceps kyushuensis* contains the BGCs for cordycepin and pentostatin. These compounds function synergistically (pentostatin inhibits adenosine deaminase, thereby protecting cordycepin from degradation), and the clustering of their respective BGCs ensures their synchronized production [92]. The aforementioned identification of up to 64 SMGCs in the *Dictyophora indusiata* genome [40] also underscores its substantial potential for synthesizing a diverse array of secondary metabolites. Consequently, the systematic mining and annotation of SMGCs within edible fungi genomes has become a crucial strategy for predicting potential secondary metabolite repertoires and discovering novel bioactive substances.

#### 2.3.3. Gene Family Expansion/Contraction and Metabolic Diversity/Adaptability

The expansion (increase in member number) or contraction (decrease in member number) of gene families is a common phenomenon in genome evolution and a primary driver of species adaptation to different environments and the development of distinct metabolic capabilities. Expansion of specific metabolic enzyme gene families often correlates with an enhanced capacity of the species to synthesize particular types of compounds. A quintessential example is the notable expansion of the cytochrome P450 (CYP450) gene family in the *G. lucidum* genome, widely considered a key factor contributing to its ability to produce an exceptionally diverse range of triterpenoid compounds (ganoderic acids) [61]. Comparative genomic analysis between *Lyophyllum decastes* and its close relative *Hypsizygus marmoreus* revealed that subsequent to their divergence, *L. decastes* experienced the expansion of 426 gene families and the contraction of 834. These alterations in gene content are highly likely responsible for the physiological differences observed between these species, potentially including variations in metabolic pathways [48]. Intriguingly, comparative genomics of the symbiotic edible fungus *Tricholoma matsutake* and its congeneric relatives revealed that, compared to saprophytic fungi, the *T. matsutake* genome has lost parts of the tryptophan metabolism pathway but gained more genes related to iron homeostasis. This is interpreted as a genetic signature of its adaptation to a symbiotic lifestyle (with pine trees) and nutrient-limited conditions (especially nitrogen sources), demonstrating that adaptive genome evolution directly reflects the unique ecological niche and life strategy of the fungus, accompanied by distinct metabolic network remodeling [56]. Similarly, variations in gene family composition among different edible fungi species account for differences in their metabolic profiles. For instance, comparative genomic analysis of *Ganoderma leucocontextum* and *G. lucidum* (both within the same genus) identified significant differences in the gene complements involved in the biosynthetic pathways of sesquiterpenoids, triterpenoids, and polyketides, supporting the observation that they possess distinct active compound profiles [43]. Collectively, these studies illustrate that the evolutionary dynamics of gene families provide a fundamental basis for the metabolic diversity and environmental adaptability of edible fungi.

#### 2.3.4. Impact of Genetic and Epigenetic Variation on Metabolic Network Regulation

Beyond gene presence/absence and copy number variations, subtle genomic variations, such as single nucleotide polymorphisms (SNPs), small insertions/deletions (InDels), and structural variations (SVs), can directly affect the activity or stability of metabolic enzymes or the function of regulatory genes, consequently leading to changes in metabolic phenotypes. The gene mutations identified in the *C. militaris* albino mutant via resequencing [73] serve as an example of this principle. Furthermore, epigenetic modifications, including DNA methylation and histone modifications, represent another critical layer of gene expression regulation that can influence metabolic network activity. Research has indicated that the genomic DNA methylation level in a serially passaged mutant strain of *C. militaris* was significantly higher compared to the wild type. Differentially expressed genes associated with differentially methylated regions (DMRs) in this mutant were enriched in pathways such as pyruvate metabolism, glycerophospholipid metabolism, DNA replication, and N-glycan biosynthesis [39]. This finding suggests that alterations in the epigenetic landscape may constitute an important regulatory mechanism governing metabolism in edible fungi, warranting further in-depth investigation.

In conclusion, the genomes of edible fungi not only furnish the complete set of genetic elements (genes) required for constructing metabolic networks but also possess inherent structural features (e.g., gene fusions, gene clusters); undergo evolutionary dynamics (e.g., gene family expansion/contraction); and harbor internal variations (genetic and epigenetic) that profoundly shape the topology, evolutionary path, and regulatory modes of these networks. These genomic-level attributes collectively dictate the unique metabolic phenotypes, the rich array of bioactive compounds, and the environmental adaptability characteristic of edible fungi. Therefore, comprehensive genomics and comparative genomics research constitutes the essential foundation and prerequisite for systematically understanding the metabolic networks within edible fungi.

## 3. Transcriptomic Analysis: Revealing the Dynamic Expression Profile of Metabolic Activities

While the genome provides a static genetic blueprint, the transcriptome captures a dynamic snapshot of gene expression under specific temporal, spatial, or conditional contexts. The transcriptome encompasses the complete set of transcripts (primarily messenger RNA (mRNA), but also various non-coding RNAs like rRNA, tRNA, snRNA, snoRNA, miRNA, lncRNA, etc.) present in a particular cell, tissue, or organism at a specific state. Serving as a crucial bridge connecting static genomic information with dynamic cellular functional states, transcriptomics primarily investigates RNA-level gene expression patterns and their regulatory mechanisms [21]. By quantifying transcripts using high-throughput sequencing, transcriptomics provides vital information for understanding how the metabolic networks of edible fungi respond to internal developmental cues (e.g., different growth stages) and external environmental stimuli (e.g., temperature, light, nutrition, stress). As research progresses, the focus has shifted from merely describing static genome composition towards exploring how genes are dynamically regulated in response to diverse internal and external factors, which is particularly critical for understanding often tightly controlled metabolic processes [20] (Table 2).

### 3.1. Principles, Technologies, and Basic Applications of Transcriptome Sequencing in Edible Fungi Research

Transcriptome Sequencing (RNA-Seq) is the current mainstream technology for studying gene expression. Its fundamental principle involves deep sequencing of total RNA or specific RNA fractions (e.g., enriched mRNA after rRNA depletion) extracted from cells or tissues using high-throughput sequencing platforms. The resulting short reads are then aligned to a reference genome or assembled de novo to obtain expression abundance information for each gene (or transcript), typically represented by metrics like FPKM (Fragments Per Kilobase of transcript per Million mapped reads), RPKM (Reads Per Kilobase of transcript per Million mapped reads), or TPM (Transcripts Per Million). In edible fungi metabolic research, the core applications of RNA-Seq include, first, identifying Differentially Expressed Genes (DEGs) by comparing transcriptomic data across different conditions (e.g., developmental stages, tissues, environmental treatments, strains); second, performing functional enrichment analyses (e.g., Gene Ontology (GO) and Kyoto Encyclopedia of Genes and Genomes (KEGG) pathway enrichment) on these DEGs using bioinformatics tools to infer which biological processes or metabolic pathways exhibit significantly altered activity under the compared conditions; third, discovering novel transcripts beyond known coding genes, including alternative splicing isoforms and non-coding RNAs potentially playing roles in metabolic regulation, such as long non-coding RNAs (lncRNAs) [148] and small non-coding RNAs (sncRNAs, including miRNAs) [149]. Although the specific functional mechanisms of non-coding RNAs in edible fungi metabolism require further investigation, existing studies suggest their potential importance. For instance, several long intergenic non-coding RNAs (lincRNAs) potentially involved in gene expression regulation were identified in the *Ganoderma lucidum* transcriptome [148], and sncRNAs have been found in *Pleurotus ostreatus* [149].

Beyond directly studying gene expression and regulation, transcriptomic data offer other significant applications, particularly for species lacking high-quality genomic information or when developing molecular markers is necessary. Edible fungi are highly valued for their nutritional content and unique flavors, but their extensive species diversity and complex genetic backgrounds necessitate accurate species identification and germplasm evaluation as prerequisites for industry development and basic research. Traditional morphology-based identification is often inefficient, susceptible to environmental influence, and inadequate for complex taxonomic delimitations. While genome sequencing provides the most comprehensive genetic information, it remains relatively costly and time-consuming. In this context, utilizing transcriptomic data for developing molecular markers or species identification presents an efficient and economical alternative. Researchers can mine Simple Sequence Repeats (SSRs), also known as microsatellites, from transcriptomic data. Due to their wide genomic distribution, high polymorphism, and codominant inheritance, SSRs are ideal markers for genetic diversity analysis, genetic map construction, and variety identification. By sequencing transcriptomes from mycelia and fruiting bodies of *Auricularia heimuer* and *A. polytricha*, Zhou et al. [150] not only analyzed transcriptional differences but also identified abundant SSR loci in both species’ transcriptomes. Although differing slightly in quantity and repeat motif types, their overall composition was similar, providing a basis for marker development in these related species. Similarly, potential SSR loci were successfully identified from transcriptomic data in *A. polytricha* [99] and *Morchella* spp. Researchers developed 15 pairs of genetically polymorphic SSR primers based on *Morchella* transcriptome sequences, validating the utility of transcriptomic data for molecular marker development [151].

Furthermore, RNA-Seq data can be employed for species identification, notably, using Internal Transcribed Spacer (ITS) sequences of ribosomal RNA genes. The ITS region (ITS1 and ITS2), being relatively conserved within species but highly variable between species and having moderate length (typically 350 bp and 400 bp, respectively), is widely recognized as the core DNA barcoding marker for fungi, effectively distinguishing closely related species. Southwest China, including Yunnan Province, harbors exceptionally rich wild edible fungi resources. However, cultivation difficulties and volatile market prices lead to instances of fraud and adulteration, sometimes including toxic mushrooms, posing food safety risks. To rapidly and accurately identify mushrooms in the market, studies have utilized RNA-Seq to directly obtain and analyze ITS sequences from collected samples. Through phylogenetic analysis of the sequencing results, one study not only discovered potential new edible fungi resources but also successfully identified toxic mushroom species mixed within commercial samples, thus demonstrating the feasibility of RNA-Seq as a rapid, preliminary method for identifying edible fungi species in circulation, including differentiating toxic from non-toxic ones [152]. Additionally, transcriptomics combined with amplicon sequencing (e.g., targeting bacterial 16S rRNA and fungal ITS sequences) has been used to investigate the microbial community structure in edible fungi cultivation environments and its impact on fungal growth. For example, studies on *Morchella* cultivation soil revealed that continuous cropping significantly reduces microbial diversity, particularly affecting groups potentially interacting (mutualistically or antagonistically) with *Morchella*. A one-year fallow period was shown to help restore the soil microbial community structure [153].

Therefore, RNA-Seq technology functions as more than a tool for tracking gene expression; its outputs provide a dynamic molecular lens into fungal life. The collective findings demonstrate its analytical strength in not only elucidating metabolic regulation but also in generating foundational data for molecular marker development, species identification, and ecological interaction studies. The broader implication is the multifaceted utility of transcriptomics in providing critical support for strategic goals such as germplasm conservation, food safety assurance, and the sustainable optimization of cultivation systems for edible fungi.

### 3.2. Changes in Gene Expression Profiles Reveal Dynamic Metabolic Regulation

Metabolic activity in edible fungi is not static but undergoes precise dynamic adjustments according to their life cycle progression and changes in the external environment. By capturing variations in gene expression levels under different conditions, transcriptomics paints a molecular picture of this dynamic regulation, serving as a primary tool for understanding how edible fungi adaptively allocate resources and execute specific physiological functions.

#### 3.2.1. Transcriptional Dynamics and Metabolic Adaptation Across Life Cycles and Tissue Specificity

The life cycle of edible fungi typically includes a vegetative growth phase (mycelia) and a reproductive growth phase encompassing primordium formation, fruiting body development, maturation, and spore production. Different developmental stages, and even distinct tissue parts within the same stage (e.g., pileus/cap, stipe/stalk, lamellae/gills, volva), often undertake different physiological functions, leading to distinct metabolic requirements and priorities. Transcriptomic studies have been widely applied to various edible fungi, including *Agaricus bisporus* [96], *Auricularia polytricha* [99], *Flammulina velutipes* [114,154], *Ganoderma lucidum* [115], *Lentinula edodes* [120], *Pleurotus eryngii* [132], *P. tuoliensis* [139,140], *P. ostreatus* [155], *Agaricus subrufescens* (often considered synonymous or related to *A. blazei*) [97], *Tricholoma matsutake* [156], *P. geesteranus* [138], *Wolfiporia cocos* [147], *Agrocybe aegerita* [157], and *Grifola frondose* [119,157], revealing dramatic yet ordered changes in the expression profiles of metabolism-related genes throughout their life cycles.

The transition from vegetative to reproductive growth represents a critical juncture, often accompanied by fundamental shifts in energy metabolism patterns and drastic morphological restructuring. For instance, studies on *F. velutipes* revealed that during cold-induced primordium formation from mycelia, the expression of genes related to nutrient uptake from the substrate (e.g., sugar transporter genes) tends to decrease, while genes associated with energy production (e.g., aerobic respiration genes), cell wall remodeling (e.g., chitin synthase and hydrolase genes), and stress responses become significantly more active [111]. This indicates that primordium formation is an energy-intensive process requiring mobilization of internal reserves and cellular reorganization. Similarly, comparative transcriptome analysis of *A. blazei* mycelia and primordia showed that the mycelial stage emphasizes gene expression related to carbohydrate metabolism (substrate decomposition for nutrition), transmembrane transport, and cell wall synthesis (nutrient acquisition and growth). In contrast, the primordial stage shifts focus towards stress responses, nucleic acid and protein synthesis (providing materials for rapid cell division and differentiation), and steroid biosynthesis (potentially affecting membrane fluidity or acting as signaling molecules), all associated with morphogenesis and stress tolerance [83]. Comparative transcriptomics across five developmental stages of *Lyophyllum decastes*, from vegetative mycelium to mature fruiting body, identified numerous DEGs encoding extracellular enzymes (nutrient acquisition/environmental modification), transcription factors (developmental regulation), and signaling components (responding to internal/external cues). This confirmed the complex regulatory network governing fruiting body development, and enzyme activity assays validated the correlation between differential expression and functional activity for some extracellular enzymes [125]. These transcriptomic insights are further substantiated by proteomic studies on *L. decastes*, which, for instance, confirmed the upregulation of specific laccases and quinone reductases at the protein level when the fungus is grown on lignin-rich substrates, directly linking genomic potential and transcriptional activity to functional protein execution in lignocellulose degradation (Section 4.2.1). Combined transcriptomic and metabolomic analysis of *Morchella importuna* across three stages, from vegetative growth to sclerotium formation (a dormant/storage structure and primordium precursor), also revealed key genes and metabolic pathways crucial for mycelial morphogenesis and transition, particularly highlighting dynamic changes in carbohydrate metabolism pathways such as starch/sucrose metabolism, pentose/glucuronate interconversions, and fructose/mannose metabolism [126]. The understanding of *M. importuna*’s developmental metabolism is further enriched by proteomic analyses (Table 3), which compared vegetative mycelia to sexual reproductive stages, identifying differential protein abundance in carbohydrate and amino acid metabolic pathways and highlighting the roles of specific proteins like lectins. Such integration provides a more robust picture of the molecular shifts occurring during its life cycle.

Even within the fruiting body development stage, significant tissue- or maturation-specific differences exist in gene expression and metabolic activity. For example, comparing transcriptomes from different parts (pileus, indusium, mycelial cord, stipe, volva) of *Dictyophora indusiata* revealed the highest overall gene expression levels in the pileus. DEGs associated with fruiting body differentiation (e.g., genes involved in tryptophan metabolism; tryptophan is a precursor for auxin/IAA, potentially involved in morphogenesis) were significantly enriched in the pileus, suggesting its central role in regulating formation and highlighting specialized metabolic functions among tissues [40]. Research on *Stropharia rugosoannulata* found that pathways for biosynthesis (protein, nucleic acid), glucose metabolism (energy/carbon skeletons), and amino acid metabolism remain active throughout fruiting body growth and development [168]. This study also examined post-transcriptional regulation, finding that alternative splicing events (especially intron retention and alternative 3′ splice sites) play significant roles during fruiting body development and maturation. Studies on the medicinally important *G. lucidum* indicated metabolic shifts during development: genes related to triterpene (ganoderic acid) and ergosterol (key membrane component) biosynthesis were highly expressed in young fruiting bodies. During sporulation, expression of genes involved in carbohydrate metabolism (providing energy/structure for spores, e.g., trehalose synthesis) significantly increased. In the later stages of spore release, genes encoding glycoside hydrolases (e.g., chitinases, glucanases) were upregulated, possibly related to the final maturation and modification of the spore wall [115].

Furthermore, different reproductive strategies (sexual vs. asexual) correspond to distinct gene expression profiles. Research on *C. militaris* showed that compared to its sexual phase (producing fruiting bodies and ascospores), the asexual phase (producing conidia) exhibited significantly higher upregulation of genes related to cellular metabolism, energy metabolism, and stress responses, possibly reflecting a strategy prioritizing rapid proliferation and dispersal [169]. In studies concerning fruiting body development and biosynthesis of the key medicinal compound cordycepin in *Ophiocordyceps sinensis* (a medicinal fungus, not typically edible, but studied similarly), RNA-Seq identified transcripts of genes commonly associated with fungal fruiting body development (e.g., pheromone receptors, G protein subunits, signaling kinases). It also pinpointed genes for key enzymes in the cordycepin pathway, such as adenosine kinase (ADK), 5′-nucleotidase (5′-NT), adenylate kinase (ADEK), and ribonucleotide reductase (RNR) subunits, enabling the preliminary construction of a cordycepin biosynthetic model based on this information [87].

In summary, by capturing gene expression dynamics across different stages, tissues, and reproductive modes throughout the life cycle, transcriptomic analysis provides a detailed map of shifting metabolic priorities, profoundly revealing the intricate control exerted by intrinsic developmental programs over metabolic networks.

#### 3.2.2. Environmental Regulation of Transcription and Metabolic Remodeling

The growth, development, and metabolic activities of edible fungi are governed not only by internal genetic programs but are also constantly influenced by external environmental factors such as temperature, humidity, light, pH, nutrient status, gas composition, and biotic stress. When environmental conditions change, particularly under unfavorable stress conditions, edible fungi initiate stress response mechanisms, remodeling their metabolic networks through regulated gene expression to adapt and survive. Transcriptomics serves as the core tool for investigating the gene expression regulation underlying this adaptive metabolic remodeling.

Temperature is a critical environmental factor influencing growth and yield. Studies on *F. velutipes* revealed significant differences in transcriptional responses to heat stress among varieties (e.g., white vs. yellow), potentially correlating with variations in heat tolerance [112]. Conversely, under cold stress or cold induction conditions, transcriptomes undergo drastic changes. For instance, studies on *V. volvacea* under cold stress [170] and *F. velutipes* during cold-induced primordium formation [111] both identified significant alterations in genes related to stress response (e.g., heat shock proteins), signal transduction, energy metabolism (adjusting respiration/fermentation), and carbohydrate metabolism (accumulating protective sugars like trehalose). Transcriptome analysis of *S. rugosoannulata* under cold stress demonstrated a general downregulation of carbohydrate utilization enzyme genes and upregulation of antioxidant enzyme (SOD, CAT) and heat shock protein (Hsp) genes, regardless of strain sensitivity. This suggests a relatively conserved molecular mechanism for cold tolerance, although the intensity and efficiency of these responses may differ among strains with varying sensitivity [144]. While these transcriptomic changes suggest a conserved cold response mechanism, proteomic analyses from the same study on *S. rugosoannulata* (Table 3) also revealed tissue-specific protein expression patterns, for instance, with stipe proteins being enriched for carbon metabolism and stress response, suggesting that transcript-level stress responses manifest in distinct proteomic profiles within different parts of the organism. Similarly, multi-omics analysis of cold-treated *F. velutipes* mycelia revealed significant enrichment of pathways related to carbohydrate metabolism, unveiling its molecular strategy for coping with cold stress [171]. Following heat stress treatment of *P. ostreatus*, researchers specifically examined the expression changes within its large Zn(II)2Cys6 zinc cluster protein-encoding gene family (often transcription factors). They found that members from different clusters exhibited distinct expression patterns (up- or downregulation), implying diverse roles within the transcriptional regulatory network responding to heat stress [85].

Light is another crucial environmental factor influencing growth and development, especially fruiting body morphogenesis and pigmentation. Research on *L. edodes* demonstrated that blue light induces changes in numerous genes. Comparing transcriptomes under blue light versus dark conditions identified 762 DEGs. Several genes validated as upregulated under blue light included those involved in morphogenesis (encoding HSPs, fibronectin domain proteins, carbohydrate esterases) and light sensing (encoding photoreceptor-related proteins with FAD/NAD-binding domains) [121]. This indicates that light signals can modulate metabolic activity and developmental processes in shiitake by activating specific gene expression. Another study simulating intense sunlight exposure found significantly enhanced expression of genes involved in DNA repair, heat shock response, and potentially photoprotective secondary metabolic pathways in *L. edodes* samples exposed for 30 and 60 min, revealing specific molecular defense mechanisms against light radiation stress [172].

Heavy metal contamination represents a potential stressor in agricultural environments. To understand fungal responses, the transcriptome of *Hypomyces chrysospermus*, a fungus known to potentially contaminate edible fungi cultivation settings, was analyzed under cadmium stress. DEGs were primarily enriched in pathways related to protein synthesis (translation); amino acid metabolism; transport and catabolism; carbohydrate metabolism; and protein folding, sorting, and degradation. Notably, upregulation was observed for genes encoding various transporters (potentially for cadmium efflux or sequestration), antioxidant enzymes (counteracting cadmium-induced oxidative stress), and detoxification enzymes (e.g., glutathione S-transferases), elucidating complex molecular mechanisms for heavy metal tolerance and detoxification [173]. This research has implications for evaluating the safety of edible fungi grown in contaminated areas and exploring the potential of fungi in bioremediation.

Biotic stress, such as infection by pathogenic fungi, is a prevalent issue in edible fungi production. Understanding fungal responses to pathogens is essential for developing resistant varieties and control strategies. For instance, investigating the interaction and pathogenesis of *Paecilomyces penicillatus* on *M. importuna*, researchers analyzed RNA-Seq data from transcripts isolated at the interaction zone. They observed dynamic changes in *M. importuna* gene expression across different infection stages. Some potentially resistance-related genes, encoding glycosyltransferase family 31 proteins and ferric chelate reductases, exhibited initial upregulation followed by downregulation. Conversely, genes encoding mannitol-1-phosphate 5-dehydrogenase (mannitol is a key osmolyte/antioxidant in some fungi) and the glucose-repressible protein Grg1 showed an opposite trend. Intriguingly, pathways related to fatty acid metabolism and biosynthesis (including unsaturated fatty acids) were significantly upregulated early in the infection, suggesting a potential role for fatty acid metabolism in fungal interactions (e.g., defense or signaling) [174]. While this study focused on transcriptomic and genomic changes, complementary proteomic analyses of *M. importuna* under such biotic stress would further elucidate the functional protein effectors involved in defense and interaction, bridging the gap between gene expression and actual protein-mediated responses. For instance, proteomic studies on *M. sextelata* have provided insights into pathogen defense (Section 5.1.3). This study provides molecular insights for enhancing white mold resistance and optimizing cultivation management in *M. importuna*.

Other abiotic stresses, such as drought, and post-harvest physiological changes, like browning, also induce significant transcriptomic alterations. Transcriptome analysis of *Auricularia cornea* (potentially a variety or relative of *A. heimuer*) during drought stress and subsequent rehydration revealed distinct adaptive strategies. Drought primarily induced tyrosine metabolism (possibly related to stress-associated phenolics), caffeine metabolism, ribosome function adjustments, phagosome formation (autophagy/recycling), proline and arginine metabolism (proline as osmoprotectant), and activation of peroxisome and MAPK signaling pathways. During rehydration, pathways activated included diterpenoid biosynthesis (potentially related to growth recovery), butyrate metabolism (GABA production, potentially stress recovery signaling), C5-branched dibasic acid metabolism, and aflatoxin biosynthesis (possibly related to interaction or stress) [88]. Addressing post-harvest browning in *A. bisporus*, comparative transcriptomics combined with metabolomics on varieties differing in browning sensitivity identified differential expression of key enzyme genes (e.g., polyphenol oxidase, PPO) and correlated these changes with the accumulation levels of relevant metabolites (e.g., organic acids, trehalose), which collectively influence browning resistance [93].

In summary, by comprehensively analyzing gene expression profile changes in response to various environmental challenges (biotic and abiotic), transcriptomic studies clearly illustrate how edible fungi flexibly remodel their metabolic networks through transcriptional regulation. This adaptation allows them to cope with changing environments, ensure survival, or execute specific physiological processes. This knowledge is invaluable for understanding ecological adaptability, optimizing cultivation conditions, enhancing stress tolerance, and improving post-harvest quality.

#### 3.2.3. Differential Gene Expression Between Strains/Varieties and Comparison of Metabolic Capabilities

Significant variations often exist between different strains or cultivated varieties of the same edible fungi species concerning growth rate, yield, fruiting body morphology, color, flavor, nutrient content, stress resistance (e.g., heat, cold, disease tolerance), and enzymatic properties. Underlying these phenotypic differences frequently lie variations at the gene expression level. Comparative transcriptomics, by directly comparing the gene expression profiles of strains with distinct genetic backgrounds or phenotypic traits, effectively links transcriptional-level differences to specific metabolic capabilities or biological characteristics. This approach helps unveil the molecular basis of these variations, facilitates the screening for superior strains, and aids in identifying functional genes controlling key traits. Such research has been undertaken in numerous edible fungi, comparing, for example, mycelia with different nuclear statuses (monokaryotic vs. dikaryotic) or varying mating type combinations [113,175,176,177,178], as well as contrasting commercial varieties or mutants exhibiting distinct features. Comparative analyses typically focus on the following:

Growth Rate and Nutrient Utilization Efficiency: Mycelial growth rate significantly impacts the production cycle length. By comparing commercial strains of *Volvariella volvacea* with differing growth rates, Liu et al. [146] found that faster-growing strains generally exhibited higher expression levels of genes related to carbohydrate utilization (e.g., encoding glycoside hydrolases, sugar transporters). This indicates that efficient nutrient uptake and decomposition capabilities are among the key factors supporting rapid growth.

Fruiting Body Quality, Flavor, and Appearance: Fruiting body quality is central to the commercial value. Transcriptome analysis of different *A. bisporus* varieties (e.g., browning-susceptible vs. resistant) across developmental stages helps identify important DEGs associated with quality formation (e.g., differential PPO expression controlling browning [93]), morphogenesis, and maturation processes [94]. The ability of *L. edodes* mycelium to undergo normal browning (transition from white to brown, necessary for subsequent fruiting) is a critical production issue. Tang et al. [179] compared transcriptomes of browning and non-browning shiitake strains, identifying expression differences in several transcription factors that potentially regulate pigment synthesis or related metabolic pathways, thus influencing the browning process. Addressing cap color diversity in *P. ostreatus*, Hou et al. [136] linked color variation to transcriptomic changes, proposing that H_2_O_2_ might act as a signal, influencing pigment accumulation by differentially regulating genes associated with the oxidative phosphorylation pathway. The texture and commercial value of *A. heimuer* relate to fruiting body wrinkling. Zhao et al. [98] compared gene expression profiles of varieties with different wrinkling characteristics, discovering that non-wrinkled varieties exhibited unique patterns in certain gene functions (potentially involving cell wall synthesis, turgor regulation) and overall expression levels, providing clues to the molecular basis of these morphological differences.

Key Enzyme Activity Differences and Regulation: Certain enzymes produced by edible fungi, such as laccases, possess significant industrial value. To identify high-laccase-yielding strains or understand their regulation, Lei et al. [122] performed comparative transcriptome analysis on *L. edodes* monokaryotic mycelia with differing laccase activities. Results revealed significant expression differences in genes related to lignin degradation (a primary laccase function), cytochrome P450-mediated metabolism (potentially substrate conversion or secondary metabolism), and carbohydrate metabolism, offering potential targets for screening high-yielding strains or enhancing laccase production by modulating related pathways.

Gene Function Validation and Regulatory Network Analysis: Comparative transcriptomics also serves as a vital tool for validating specific gene functions. By constructing overexpression (OE) or gene silencing (e.g., RNAi) strains for a target gene and comparing their transcriptomes with wild-type or control strains, the downstream regulatory network of the gene can be elucidated. For instance, Cao et al. [133] investigated the function of a G protein α-subunit gene (PeGNAI) in *P. eryngii*. Comparing transcriptomes of PeGNAI OE and RNAi strains demonstrated that altered expression levels significantly impacted genes in pathways related to amylase and laccase activities, thereby confirming the role of PeGNAI in regulating growth, development, and associated enzyme activities in king oyster mushroom.

Exploring Superior Germplasm Resources and Assisting Breeding: Comparing transcriptomes of wild germplasm versus cultivated varieties, or strains from different geographic origins or with varying stress tolerance levels, can identify specific genes or regulatory modules associated with desirable traits (e.g., high nutritional value, strong stress resistance, unique flavor). This provides candidate targets for molecular marker-assisted breeding (MAS) or direct genetic improvement.

In conclusion, employing transcriptomic techniques to compare gene expression differences between edible fungi strains or varieties constitutes a powerful research strategy. It not only aids in understanding the molecular origins of biological trait diversity, correlating specific metabolic capabilities or phenotypes with gene expression patterns, but also effectively facilitates the screening of strains possessing superior characteristics and the discovery of core genes regulating key pathways. This provides a robust theoretical basis and technical support for the genetic improvement and germplasm innovation of edible fungi.

## 4. Proteomic Research: Directly Addressing the Functional Execution Level of Metabolism

While the genome provides the genetic blueprint and the transcriptome reveals gene expression dynamics, proteomics offers direct insights into the proteome—the functional executors of cellular processes. Understanding the proteome is crucial, as it directly reflects metabolic activities and provides a critical layer for validating and complementing genomic and transcriptomic findings, particularly in the complex metabolic networks of edible fungi. The proteome refers to the entire complement of proteins expressed by a cell, tissue, or organism at a specific time point and under specific conditions [20,22]. Proteomics is the discipline dedicated to the systematic study of protein composition, abundance, post-translational modifications (PTMs), subcellular localization, and protein–protein interactions (PPIs) [22]. As proteins are the direct effectors of biological function, proteomic research offers information closer to the actual functional state of an organism compared to genomics and transcriptomics. Particularly for understanding complex metabolic activities, it directly detects the enzymes performing metabolic reactions and the proteins regulating these processes [23]. Consequently, proteomics is regarded as an essential complement and validation for genomic and transcriptomic studies, crucial for the comprehensive elucidation of metabolic networks and their regulatory mechanisms in edible fungi (Table 3).

### 4.1. Overview of Proteomic Research Methods

Currently, the most commonly employed core technologies in edible fungi proteomics are based on mass spectrometry (MS). Depending on the sample separation and quantification strategies, these can be broadly categorized into two main types:

Gel-based proteomics: Two-dimensional gel electrophoresis (2-DE) is the classic method within this category. The method 2-DE separates complex protein mixtures on a gel based on two properties, isoelectric point (pI) and molecular weight (MW), generating a protein spot map. By comparing protein spot patterns between different conditions (e.g., treatments, developmental stages), differentially expressed proteins can be initially screened. Subsequently, protein spots of interest are excised from the gel, enzymatically digested (typically with trypsin), and the resulting peptide mixtures are identified using MS techniques (e.g., MALDI-TOF MS or LC-ESI-MS/MS) [26]. The advantages of 2-DE include its ability to visualize changes in protein abundance intuitively and detect intact proteins with their major PTMs. However, its throughput is relatively low, detection sensitivity for low-abundance proteins and those with extreme pI/MW values is limited, the procedure is relatively laborious, and quantitative accuracy can be constrained.

Gel-free/Shotgun proteomics (LC-MS/MS based): This approach involves enzymatically digesting the protein sample into a peptide mixture first, followed by separation using high-performance liquid chromatography (HPLC, typically reversed-phase LC, RPLC). The separated peptides are directly introduced into the mass spectrometer for analysis and identification. Based on the quantification method, LC-MS/MS techniques can be further subdivided into label-based and label-free quantification (LFQ) methods. The former (label-based quantification) includes techniques such as iTRAQ (Isobaric Tags for Relative and Absolute Quantitation) and TMT (Tandem Mass Tags). These techniques utilize isotopically labeled chemical reagents to tag peptides from different samples (allowing for multiplexing, e.g., simultaneous comparison of up to 8 or 16 samples with iTRAQ or TMT, respectively). At the MS1 level (first mass spectrometry scan), tagged versions of the same peptide originating from different samples are isobaric (exhibit identical mass-to-charge ratios, *m*/*z*) and thus indistinguishable. However, upon fragmentation during tandem mass spectrometry (MS/MS), the tags yield reporter ions with distinct m/z values. Measuring the intensity ratios of these reporter ions enables the relative quantification of proteins across the different samples [26]. Label-based quantification offers high throughput and generally good quantitative accuracy. The latter (label-free quantification) does not require chemical labeling of peptides. Instead, relative quantification is achieved by comparing peptide signal intensities (e.g., peak areas or ion currents) or spectral counts detected during LC-MS/MS analysis across different samples. Label-free methods offer a simpler workflow, are not limited by multiplexing sample numbers, are comparatively less expensive, and can potentially cover a wider protein dynamic range [180]. However, achieving high quantitative accuracy and reproducibility places greater demands on experimental consistency and sophisticated data analysis workflows.

In edible fungi research, Differential Proteomics is the most widely applied strategy. Whether using 2-DE or LC-MS/MS, the core objective is to compare proteome expression profiles under different physiological states (e.g., developmental stages, tissues/organs), environmental conditions (e.g., stress treatments, cultivation substrates), or genetic backgrounds (e.g., different varieties, mutants). This allows for the screening of key Differentially Expressed Proteins (DEPs), particularly enzymes and regulatory proteins associated with metabolic pathways [26], providing direct functional-level evidence for the molecular mechanisms underlying specific biological processes. Recently, the scope of proteomics in edible fungi research has strategically expanded towards identifying medically relevant proteins, signifying a shift to explore bioactive potentials beyond nutrition [23]. A critical analytical challenge, however, remains: the often observed discrepancies between transcript and protein abundances, and the still limited coverage of the full proteome, especially dynamic PTMs, complicate direct gene-to-function inferences [181]. The broader implication is that, while proteomics provides invaluable functional data, its analytical power is maximized when integrated with other omics to navigate these complexities.

### 4.2. Protein Expression Abundance and Functional Characterization in Metabolic Analysis

By directly measuring the types and abundance changes in intracellular proteins, proteomics provides the most direct evidence for understanding which functional modules are activated and which metabolic pathways are operative during specific physiological processes. It can validate predictions made at the transcript level, reveal regulation occurring at the translational and post-translational levels, and elucidate the dynamic changes in proteins as they execute specific functions.

#### 4.2.1. Protein-Level Analysis and Functional Confirmation of Important Enzymes

Enzymes are the core executors of metabolic networks. Proteomic techniques have been extensively used to directly detect changes in the abundance of enzyme proteins within key metabolic pathways, thereby confirming their actual roles in specific processes.

Pathway-Specific Enzyme Confirmation: When studying specific metabolic pathways, proteomics can directly confirm the involvement and activity changes in key enzymes. For instance, investigating the response of *Lyophyllum decastes* to lignin-rich substrates involved combined genomic and proteomic analysis. Genomics predicted numerous CAZyme genes, particularly laccases. Subsequent proteomic analysis (identifying 1071 proteins, including CAZymes and secreted enzymes) revealed that the abundance of several specific laccases and quinone reductases was indeed significantly upregulated in the lignin-rich medium [48]. This result directly confirmed, at the protein level, the central role of these enzymes in lignin degradation, strongly complementing and validating the genomic predictions. Similarly, proteomic analysis of *Schizophyllum commune*, aimed at exploring its biotechnological potential, successfully identified key enzymes involved in the trehalose biosynthesis pathway, such as trehalose synthase, trehalose phosphatase, and trehalose phosphorylase [165]. As trehalose is a high-value carbohydrate with broad applications, identifying these enzymes via proteomics opens possibilities for industrial enzymatic synthesis using S. commune or its enzyme systems.

Post-Harvest Processes: Edible fungi are susceptible to autolysis, browning, and decay during post-harvest storage, processes closely linked to internal enzyme activities. Proteomics offers powerful tools to uncover the enzymatic mechanisms involved. Quantitative proteomics analysis of post-harvest autolysis in *Coprinus comatus* found that hydrolytic enzymes involved in cell wall metabolism (e.g., chitinases, β-glucanases) significantly increased at the protein level during early storage (cap opening), regulated by ribosomal activity. This led to cell wall polysaccharide degradation, structural disruption, and ultimately autolysis. The abundance of these enzymes then rapidly declined later in the process. Concurrently, accumulation of reactive oxygen species (ROS) from respiratory metabolism throughout storage caused oxidative stress, activating MAPK signaling cascades in later stages, potentially inducing apoptosis and accelerating autolysis [182]. Using label-free proteomics to study *Flammulina velutipes* under commercial modified atmosphere packaging (MAP; low O_2_, high CO_2_) revealed significant enrichment of the tricarboxylic acid (TCA) cycle pathway mid-storage. The protein abundance of key enzymes like citrate synthase (CS) increased, indicating attempts to maintain energy supply against storage stress. (Note: AAS mentioned in the original text might be misidentified or mislabeled, potentially referring to other amino acid metabolism or TCA cycle enzymes). Towards the end of storage, antibiotic biosynthesis became prominent, alongside changes in carbon and amino acid metabolism, such as a significant decrease in the glycolytic enzyme triosephosphate isomerase (TPI) protein abundance [180]. These complex changes in the enzyme protein abundance collectively reflect the sophisticated energy metabolism regulation employed by F. velutipes to sustain viability during storage.

Environmental Stress Responses: Environmental stresses also trigger dramatic responses at the enzyme protein level. Proteomic analysis of frozen *Auricularia heimuer* mycelia directly quantified increased levels of antioxidant enzymes like superoxide dismutase (SOD) and peroxidase (POD), indicating cellular activation of defense mechanisms against freezing-induced oxidative stress. The study also found that freezing inhibited the activity of tyrosinase, a key enzyme in melanin synthesis, while enhancing laccase activity [158], revealing complex direct effects of cold stress on the melanin pathway. High CO_2_ concentrations can be encountered during cultivation or storage of some species like *Pleurotus ostreatus*. Comparative proteomics of *P. ostreatus* fruiting bodies grown under high CO_2_ revealed reduced metabolic activity related to kinases (involved in signaling and metabolic regulation) and a decreased abundance of several elongation factors involved in protein synthesis. Additionally, expression of proteins related to cell wall synthesis and sexual differentiation processes was significantly affected [183]. These results unveil, at the protein execution level, the specific regulatory mechanisms by which high CO_2_ impacts *P. ostreatus* growth and metabolism.

In summary, proteomics provides “seeing is believing” evidence by directly measuring and comparing enzyme protein abundance. This validates transcript-level predictions and can uncover the impact of post-transcriptional regulation on enzyme levels, thereby more accurately elucidating the actual roles and importance of specific enzymes in the physiological activities and stress responses of edible fungi.

#### 4.2.2. Global Proteome Responses and Metabolic Correlations Under Different Physiological States

Beyond focusing on specific enzymes, the power of proteomics lies in its ability to provide a global perspective. By comparing the overall proteome composition and abundance changes in edible fungi across different developmental stages, tissues, environmental conditions, or morphologies, it can depict the complete metabolic landscape and functional priorities under specific physiological states.

Developmental Dynamics: Fungal development is a continuous, complex process involving significant morphological changes and physiological transitions, inevitably reflected in proteome dynamics. iTRAQ-based quantitative proteomic analysis of *Volvariella volvacea* at different time points (0, 24, 60, 96 h) in post-harvest storage at 15 °C showed a significant decline in the abundance of proteins associated with stress response and cellular homeostasis (e.g., stress-induced PAB1, membrane integrity protein RPG1) as storage progressed. Proteins involved in gene expression regulation (RNA transcription-related) and the protein synthesis machinery (all differentially expressed ribosomal proteins) were also generally downregulated. Conversely, some proteins related to fatty acid metabolism showed an upward trend, potentially further affecting membrane function [167]. These global proteomic trends collectively depict a senescence process in stored *V. volvacea*, characterized by diminishing stress resistance, declining cellular structural stability, and attenuated overall metabolic activity (especially anabolism). Using label-free LC-MS/MS, research on the commercially valuable *Hypsizygus marmoreus* (white beech mushroom) compared protein expression profiles across developmental stages (pre-primordium to primordium emergence), identifying various CAZymes, transcription factors, and heat shock proteins with altered abundance [160]. Further studies examined three consecutive stages from mycelial physical treatment (inducing primordia) to primordium appearance, finding continuous evolution in differential protein expression involving various functional proteins like importins, dehydrogenases, and HSPs [159]. This highlights the capability of proteomics to map dynamic protein changes during development, potentially reflecting shifting metabolic demands, such as increased carbohydrate metabolism enzymes needed during rapid growth phases. Building upon the transcriptomic insights into *M. importuna* development (Section 3.2.1), proteomic comparisons between its vegetative mycelia and sexual reproductive stages further elucidated the molecular underpinnings. These proteomic studies revealed significant enrichment in protein abundance related to ribosome biogenesis in the reproductive stages [163], suggesting that the observed transcriptomic shifts translate into increased protein synthesis machinery to support rapid fruiting body development. iTRAQ comparative proteomics across different growth stages of *Ophiocordyceps sinensis* identified several differentially abundant proteins (DAPs) shared across stages, involved in pathways like carbon transport/metabolism and antioxidant activity, providing protein-level clues to its unique growth characteristics and medicinal compound synthesis [164]. Proteomic study of *Pleurotus giganteus* reported for the first time an upregulation of proteins associated with carbon metabolism and energy generation pathways in mature fruiting bodies, consistent with findings in other mushrooms, further confirming the dominant role of carbohydrate catabolism in supporting rapid growth and substance accumulation in fruiting bodies [184]. Beyond developmental stages, proteomics also dissects functional differences between distinct morphologies of the same species. For example, *Tremella fuciformis* exists in yeast-like and filamentous forms. Comparative proteomics (combining global and targeted approaches) of its dikaryotic yeast-like basidiospores (FBMds) and dikaryotic mycelia (DM) indicated that although the overall basal synthetic and metabolic activity might be lower in DM, the abundance and phosphorylation levels of certain signaling proteins (e.g., MAPK pathway components) were upregulated [166]. This suggests distinct signaling strategies might be employed in different morphologies to adapt to specific lifestyles or environments, revealing significant differences in synthesis, metabolic activity, and signaling responses between the two forms.

Environmental Adaptation and Cultivation Effects: Environmental adaptability is key to the survival and distribution of edible fungi. Proteomics has been used to investigate responses to different geographical environments or cultivation conditions. A proteomic study (validated with transcriptomic data) on fruiting bodies of the prized white truffle (*Tuber magnatum Pico*) from different Italian regions (North, Center, South) found significant geographic variation in the abundance of key enzymes in the sulfur metabolism pathway, such as cystathionine γ-lyase [185]. This strongly suggests that white truffles might adapt to habitats with varying soil conditions (e.g., sulfur content, redox state) by dynamically regulating their sulfur metabolism network (sulfur compounds contribute significantly to truffle aroma and may also be involved in stress response). Combining genomic potential (reflected by transcriptomics) with actual protein expression levels (proteomics) allows for a more comprehensive understanding of mushroom metabolic profiles and environmental responses [55]. The cultivation substrate significantly impacts yield and quality. Integrated proteomics and metabolomics studies on *Lentinula edodes* grown on different alternative substrates (e.g., supplemented with wolfberry or Sea buckthorn) showed that these substrates significantly increased the protein and fatty acid content in fruiting bodies. Proteomic analysis revealed concordant upregulation of proteins involved in carbohydrate metabolism and oxidoreductase activity (potentially related to energy supply and stress response) in mushrooms grown on alternative substrates [162,186]. This clearly demonstrates proteomics is an effective tool to understand how cultivation conditions (like substrate composition) influence nutritional quality and the metabolic state by analyzing protein expression changes linked to key metabolic pathways. Furthermore, proteomic analyses of fruiting bodies from two other important medicinal fungi, *Sparassis crispa* (cauliflower mushroom) and *Hericium erinaceum* (lion’s mane), revealed a wealth of functionally diverse proteins, including 14-3-3 proteins involved in signal transduction and septins involved in cell division, providing deeper insight into their proteome complexity [187]. These proteomic explorations, especially when contextualized with genomic potential (e.g., Reference for *Sparassis* [54]) and transcriptomic responses (as seen in various examples in Section 3), highlight how edible fungi modulate their protein landscapes for specific functions, from environmental adaptation (e.g., *Tuber magnatum Pico*) to reflecting cultivation impacts on nutritional quality (e.g., *L. edodes*). Future focused proteomic studies, particularly on underrepresented species or delving into PTMs for common species like *L. edodes* and *P. ostreatus*, are crucial for building a more comprehensive, integrated understanding of fungal biology.

In conclusion, proteomics, by analyzing global protein changes across various conditions, offers a comprehensive and systematic understanding of the activity levels, regulatory priorities, and functional adaptations of metabolic networks in edible fungi. This provides crucial protein-level information for deepening our understanding of their biological characteristics, optimizing cultivation and management practices, and developing strategies for resource utilization.

## 5. Integrative Omics Analysis: Towards a Systems Understanding of Edible Fungi Metabolic Networks

Genomics provides the static repository of genetic information, transcriptomics reveals the dynamic changes in gene expression, and proteomics directly reflects the functional state of effector molecules. However, the relationships between these layers are not simply linear. Significant discrepancies can exist between the transcriptional level of a gene and the abundance of its encoded protein (due to factors like post-transcriptional regulation, translational control, and protein degradation), and protein abundance does not fully equate to its activity (requiring consideration of post-translational modifications, allosteric regulation, cofactor availability, etc.). Therefore, data from a single omics layer can only provide insights into specific facets of complex biological systems. To more systematically and comprehensively dissect the intricate metabolic networks of edible fungi and their sophisticated regulatory mechanisms, the integrated analysis (Integrative Omics) of genomic, transcriptomic, and proteomic data—sometimes also including metabolomic data (metabolomics, studying the global profile of small molecule metabolites)—has become an inevitable trend and forefront direction in current life science research [188]. This integrated approach helps trace the flow of information from genetic potential (genome) through transcriptional response (transcriptome) to functional execution (proteome), ultimately manifesting as metabolic output (metabolome). Such integration yields deeper and more systematic biological insights than single-omic studies can offer [20,40,189] (Figure 1, Table 4).

### 5.1. Examples of Integrated Analysis of Key Metabolic Pathways and Biological Processes

Multi-omics integration has demonstrated significant power in various aspects of edible fungi research, particularly in elucidating key metabolic pathways, understanding complex biological processes (e.g., development, stress response), and linking genotypes to phenotypes (Figure 1).

#### 5.1.1. Integrative Analysis of Bioactive Substance Biosynthetic Pathways

Bioactive compounds produced by edible fungi, such as polysaccharides, terpenoids, sterols, and nucleosides, are sources of their significant nutritional and medicinal value. Integrated analysis can more clearly delineate the biosynthetic pathways of these compounds and their regulatory networks. For example, regarding the synthesis of widely studied fungal polysaccharides (mainly β-glucans), integrating genomic annotation identifying β-glucan synthase gene family members [61,83] with transcriptomic data revealing upregulated expression patterns of these genes during specific developmental stages (e.g., *Ganoderma lucidum* sporulation [115], *Agaricus blazei* primordia [83]) or under specific induction conditions (e.g., L-phenylalanine inducing extracellular polysaccharide production in *G. lucidum* [116]), and further confirming corresponding changes in synthase protein abundance via proteomics, would collectively construct a complete regulatory map of the polysaccharide synthesis pathway from gene to functional expression. Key targets in polysaccharide biosynthesis include β-glucan synthases and chitin synthases and their regulators. Similarly, for the synthesis of terpenoids and sterols like ganoderic acids (triterpenes) and ergosterol, combining genomic identification of synthases (e.g., terpene synthases, squalene epoxidase, CYP450s) [40,61,194] with transcriptomic studies of their expression patterns in specific stages (e.g., high expression in young *G. lucidum* fruiting bodies [115]) or tissues allows for the construction of more complete and reliable biosynthetic pathway models, predicting key rate-limiting steps or regulatory nodes. In terpenoid pathways, important enzymes such as HMG-CoA reductase, squalene synthase, lanosterol synthase, and various cytochrome P450 enzymes are crucial targets. Research on *C. militaris* went further; Vongsangnak et al. [36], based on its genome sequence, comparative genomics, and the literature information (potentially implicitly using transcriptomic or proteomic data), reconstructed six core metabolic pathway networks (carbohydrate, energy, amino acid, nucleotide, lipid, cofactor). They specifically focused on the adenine metabolic pathway and cordycepin biosynthesis, relevant to its medicinal value, performing cross-species comparisons to infer the evolutionary origins of the pathway and potential reasons for high yield (e.g., deletion of ribonucleotide reductase inhibitor sequences). Nucleoside compound biosynthesis, like cordycepin, involves purine de novo synthesis and modification enzymes (e.g., adenosine kinase, SAM-cycle-related enzymes) and potentially the pentose phosphate pathway. Ergothioneine biosynthesis, relevant for fungi like *P. ostreatus*, relies on specific enzymes like EgtB and EgtD. Some fungi also produce phenolic and polyphenolic compounds with antioxidant properties via the shikimate pathway and phenylalanine ammonia-lyase (PAL). Studies on *Hericium erinaceus* also emphasize using multi-omics approaches (starting from genomics) to understand the biosynthesis of complex secondary metabolites (e.g., hericenones, erinacines) and achieve their rapid identification and quantification [204]. Beyond these, other pathways holding significant promise for metabolic engineering based on multi-omics insights include the biosynthesis of fungal polysaccharides, particularly β-glucans, which involves enzymes like β-glucan synthases and chitin synthases and their regulators, and are prized for immunomodulatory activities [61,83,115,116] [Section 5.1.1]. Additionally, phenolic and polyphenolic compound biosynthesis pathways (via the shikimate pathway, PAL) are targets for enhancing antioxidant properties. The ergothioneine biosynthesis pathway, involving enzymes like EgtB and EgtD, is also a key target in mushrooms like *P. ostreatus* and *Agaricus bisporus* due to ergothioneine’s potent antioxidant capabilities. Integrated omics approaches are pivotal for identifying rate-limiting steps and regulatory control points within these complex pathways to guide effective engineering strategies.

#### 5.1.2. Integrated Regulation of Fruiting Body Development and Differentiation

Fruiting body formation is a critical event in the life cycle of edible fungi and the primary source of their edible and medicinal value. This process is controlled by complex gene regulatory networks. Multi-omics integration helps reveal the underlying molecular mechanisms. The study on *Dictyophora indusiata* [40] serves as a compelling illustration of how multi-omics integration (genomics, transcriptomics, and metabolomics) can unravel complex biological processes like fruiting body differentiation, moving beyond descriptive findings to mechanistic insights. Genomic analysis first established the genetic potential, revealing numerous terpene synthesis gene clusters potentially linked to its unique characteristics. Transcriptomics then pinpointed the pileus as a hub of gene activity, with significant upregulation of tryptophan metabolism pathway genes. Crucially, metabolomics provided functional validation by demonstrating the differential accumulation of tryptophan-derived metabolites, such as auxin (IAA), across tissues. This integrated approach did not just correlate disparate datasets but forged a cohesive molecular narrative: from the genetic blueprint (gene clusters), through dynamic transcriptional regulation (tissue-specific pathway activation), to the functional metabolic output (auxin accumulation). Such integrated evidence strongly supports a model where spatially regulated tryptophan metabolism plays a pivotal role in controlling differentiation processes within the fruiting body, a significantly deeper insight than any single-omics approach could have provided alone. Similarly, research on *Pleurotus giganteus* integrated transcriptomic, proteomic, and nutritional composition data, revealing bidirectional transport of substrates (nutrients) and mRNA molecules between the pileus and stipe during fruiting body development [184]. This highlights complex interactions and coordination mechanisms in gene expression, protein synthesis, and nutrient allocation between different parts of the fruiting body, offering new perspectives on the physiological processes controlling growth and morphogenesis. Combined transcriptomic and proteomic analysis of *Morchella sextelata* from the young to harvested stages, using GO and KEGG enrichment, identified significant differences in the expression of genes and proteins involved in metabolic pathways (especially sugar metabolism) between the two stages, depicting the metabolic shifts during fruiting body maturation [205].

#### 5.1.3. Systematic Analysis of Stress Response Mechanisms

Edible fungi frequently encounter various stresses in natural or cultivated environments, such as temperature fluctuations or pathogen attacks. Integrated analysis facilitates a more comprehensive understanding of the complex regulatory networks underlying their stress responses. Integrated transcriptomic and proteomic analysis of *M. sextelata* strains with different heat tolerances under heat shock conditions not only identified conserved heat shock response patterns across all strains but also revealed heterogeneous response strategies among strains with varying thermotolerance. Based on these data, the researchers proposed the first molecular regulatory network model for heat shock adaptation in *M. sextelata*, providing a new framework for understanding thermotolerance mechanisms in large ascomycetes [200]. Also focusing on *M. sextelata*, another study integrated transcriptomic and proteomic data to analyze its response mechanism to infection by a pathogenic fungus (*Pseudodiploöspora longispora*). Results showed that pathways related to oxidoreductase activity, peroxisome function, lipid transport and metabolism, cell wall biogenesis, and cell membrane composition were significantly enriched or altered at the transcriptional and/or protein levels post-infection [189]. This clearly demonstrates the power of integrated omics in uncovering complex interactions between edible fungi and pathogens and elucidating the molecular mechanisms of their defense responses. Regarding post-harvest physiological changes, such as deterioration of *Pleurotus tuoliensis* during storage, Wu et al. [188] successfully employed integrated transcriptomic and proteomic analysis. They linked the upregulation of CAZyme gene expression (e.g., laccase, chitinase, glucanase) at the transcript level to increased protein abundance of these enzymes and observed fruiting body softening. Concurrently, they associated the upregulation of respiration-related enzymes (e.g., CS, ICDH, mitochondrial respiratory complex subunits) at both transcript and protein levels with increased ROS production and the response of the antioxidant system (e.g., Mn-SOD gene upregulation, Hsp60 protein upregulation possibly interacting with it), thus systematically dissecting the molecular events involved in post-harvest deterioration and their interrelationships. It is also important to acknowledge a common limitation in many current stress response studies: conventional ‘bulk’ omics analyses often average out molecular responses from different parts of the fungus (mycelia, cap, stipe) or even heterogeneous cell populations within the same tissue. This can mask the spatial specificity of metabolic regulation during stress. Future integration efforts could benefit significantly from spatial omics or single-cell omics technologies, although their application in edible fungi is currently limited, to achieve a more granular understanding of these responses.

#### 5.1.4. Association Between Optimized Cultivation Conditions and Quality Attributes

Integrated analysis can also be used to understand how cultivation conditions, such as the substrate, influence the metabolism and quality of edible fungi. Integrated proteomics and metabolomics studies on *L. edodes* cultivated on different alternative substrates (supplemented with wolfberry or Sea buckthorn) successfully linked observed proteomic changes (e.g., upregulation of carbohydrate and oxidoreductase-related proteins) directly to alterations in the metabolite profile (e.g., increased protein and fatty acid content). This provided deeper insights into how substrate composition affects the nutritional quality of shiitake by modulating specific metabolic pathways [162]. This type of integrated analysis holds significant practical value for guiding breeding efforts (selecting strains that utilize specific substrates more efficiently) and optimizing cultivation processes (designing substrate formulas that promote the accumulation of target nutrients or bioactive compounds).

### 5.2. Challenges and Methods of Integrative Analysis

Despite the promising outlook for multi-omics integration, its practical application faces numerous challenges (Figure 1). First is persistent data heterogeneity and scale differences: different omics data types (gene sequences, RNA abundance, protein abundance, metabolite concentrations) possess distinct characteristics, distributions, dynamic ranges, and scales, making effective data standardization, weighted integration, and direct comparison problematic. Recent progress underscores that proteomic data, especially those involving post-translational modifications (PTMs), still exhibit a significant gap in dynamic range and coverage compared to transcriptomic data, making the linear correlation analysis of “gene-transcript-protein-function” particularly challenging [181]. Second is the complexity of biological time lags and feedback regulation: significant delays exist from gene transcription to protein translation, and further to protein function impacting metabolite levels. Moreover, complex intracellular feedback loops (e.g., protein degradation regulation, metabolite feedback inhibition of upstream gene expression) can mislead integration analyses based on simple correlations, complicating the establishment of causal relationships between different omics datasets. Third are limitations in functional annotation: the genomes of many edible fungi remain incompletely annotated, with numerous genes of unknown function (“hypothetical proteins”), which directly limits the functional interpretation and integration of transcriptomic and proteomic data [Section 2.2]. Fourth is spatial heterogeneity: differences in mycelia, various parts of the fruiting body, and even cellular heterogeneity within the same tissue are often averaged out in conventional “bulk” omics analyses, failing to accurately reflect the spatial specificity of metabolic regulation, an issue that emerging single-cell omics technologies aim to address [Section 6]. Fifth is the applicability of computational and statistical models: many existing multi-omics integration algorithms were initially developed for model organisms, and their direct application to edible fungi might not fully account for unique fungal biological characteristics, such as heterokaryosis or complex secondary metabolic networks. Finally, there are issues of data completeness and accuracy, particularly at the proteomic and metabolomic levels, where current technologies often fall short of comprehensive coverage and precise quantification for all molecules. To address these challenges, researchers have developed a range of bioinformatics methods and tools. Common strategies include the following:

Pathway-based integration: Utilizing public pathway databases like KEGG and MetaCyc, different omics data are mapped onto known metabolic or signaling pathways. Beyond KEGG and MetaCyc, valuable resources for fungal researchers include FungiDB (VEuPathDB), which consolidates genomic, transcriptomic, and some proteomic data, supporting comparative analyses (https://fungidb.org/fungidb/ accessed on 14 April 2025). MycoCosm by JGI is a crucial repository for fungal genome sequences and annotations (https://mycocosm.jgi.doe.gov/mycocosm/home accessed on 14 April 2025). The STRING database remains useful for predicting and visualizing protein–protein interaction networks (https://string-db.org/ accessed on 14 April 2025). Additionally, specialized databases like the Chinese Ganoderma Lucidum Database (https://nmdc.cn/resource/db/linzhiitem accessed on 14 April 2025) can provide targeted information for specific genera. Analysis then focuses on overall pathway activity changes or identifies pathway nodes (genes/proteins/metabolites) exhibiting significant alterations across multiple omics levels.

Network-based integration: This involves constructing gene regulatory networks, protein–protein interaction (PPI) networks, or metabolic networks, and then integrating various omics data into these models. Analysis focuses on changes in network topology, identifying key regulatory hubs or functional modules. Network visualization tools like Cytoscape (latest stable version from https://cytoscape.org, accessed 14 April 2025) play a vital role here. More advanced network-based methods might integrate Weighted Gene Co-expression Network Analysis (WGCNA) to identify cross-omics functional modules. For PPI, the STRING database is a useful resource.

Multivariate statistical methods: Techniques such as Partial Least Squares Discriminant Analysis (PLS-DA), Canonical Correlation Analysis (CCA), and Multi-block analysis can handle multiple high-dimensional datasets, seeking correlation patterns or common trends among different omics data. Multi-omics data integration platforms (e.g., the mixOmics R package (https://mixomics.org/ on 14 April 2025), functionalities within the MetaboAnalyst website (https://metaboanalyst.ca/ on 14 April 2025)) provide tools for these analyses. Recent developments also include deep learning-based multi-omics integration models for identifying non-linear relationships and predicting phenotypes [206].

Mathematical modeling: This particularly utilizes Genome-Scale Metabolic Models (GSMMs). Based on genomic annotation and stoichiometric principles, GSMMs are mathematical representations of all known metabolic reactions in an organism, including their gene–protein-reaction (GPR) associations. By integrating transcriptomic or proteomic data to constrain reaction rates (fluxes) within the model, GSMMs can predict cellular metabolic phenotypes (e.g., growth rates, byproduct secretion rates, metabolic flux distributions) under specific conditions. Representing one of the most advanced forms of integration, GSMMs enable prediction from genotype to phenotype, but their application in edible fungi research is still nascent, though recent progress includes the development and application of GSMMs for species like *C. militaris* using transcriptomic and exometabolomic data for flux balance analysis [207,208]. Other useful databases include FungiDB (VEuPathDB) for broad fungal omics data, MycoCosm (JGI) for fungal genomes, and potentially species-specific databases like the Chinese Ganoderma Lucidum Database. General integration platforms like OmicsIntegrator or functionalities within Galaxy and Tbtools-II may also assist researchers [36].

Addressing these multifaceted challenges necessitates the development of bespoke integration strategies and bioinformatics tools attuned to the unique biology of edible fungi, such as their complex life cycles and nuclear states. The broader implication of such advancements is transformative: successfully integrating multi-omics data (genomics, transcriptomics, proteomics, and often metabolomics) will shift the research paradigm from component-level analysis to a systems-level understanding of metabolic network complexity and regulation. Ultimately, this analytical leap is foundational for enabling the rational design, predictive modeling, and targeted optimization of fungal metabolic processes, offering insights far exceeding the sum of individual omic parts and paving the way for novel biotechnological applications.

## 6. Summary

Edible fungi, a treasured biological resource, offer diverse flavors and significant nutritional and medicinal benefits owing to their rich bioactive compounds like nucleosides, polysaccharides, terpenoids, and sterols, positioning them prominently in health foods and biopharmaceuticals. Growing consumer demand for enhanced quality, yield, and specific bioactives, alongside challenges such as stringent cultivation needs for superior varieties, high production costs, germplasm scarcity, and post-harvest spoilage, necessitates a profound understanding of their complex metabolic processes.

**Figure 2:**
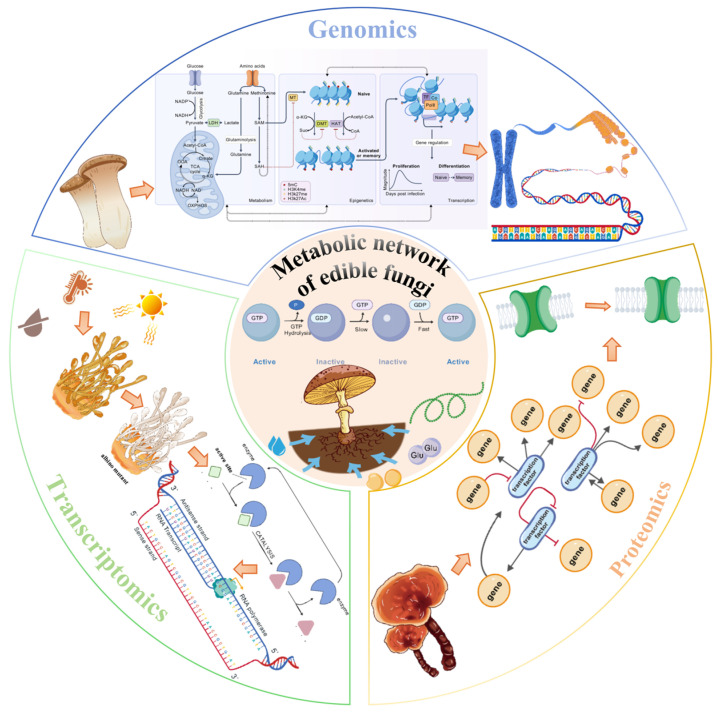
Application of omics technologies in understanding the metabolic network of edible fungi. This diagram illustrates how genomics provides the blueprint, transcriptomics reveals expression dynamics, and proteomics identifies functional protein effectors, all contributing to the elucidation and potential manipulation of metabolic pathways and cellular processes in edible fungi (created with BioGDP.com).

The “classical triad” of omics—genomics, transcriptomics, and proteomics—has been pivotal in advancing this understanding (Figure 2). Genomics furnishes the genetic blueprint, revealing metabolic gene repertoires and evolutionary signatures [20,49,50,51,66,81]. Transcriptomics demonstrates dynamic gene expression in response to developmental and environmental cues, uncovering regulatory logic [40,93,121,125,126,144,168,171]. Proteomics directly observes the execution of metabolic functions, validating predictions and revealing the impact of translational and post-translational regulation on metabolic activity [23,48,55,159,162,165,186,187]. These technologies, applied independently or through integrated approaches, have significantly advanced our comprehension of core metabolic pathways (e.g., carbohydrate, energy, amino acid metabolism) and the biosynthetic pathways of important secondary metabolites (e.g., polysaccharides, terpenoids, cordycepin), along with their intricate regulatory mechanisms. This collective knowledge forms the bedrock for future innovations in the edible fungi industry (Figure 1).

## 7. Future Directions, Challenges, and Translational Potential

The burgeoning knowledge derived from omics studies is progressively being translated into practical strategies and technologies aimed at advancing the edible fungi industry. However, significant challenges persist alongside vast potential for future research and application.

### 7.1. Translating Omics Discoveries into Applications

Guiding Precision Breeding and Genetic Improvement: Omics technologies efficiently identify genes or molecular markers [209] associated with key economic traits, such as high yield, superior quality [93], and stress resistance [111,148,154]. This information, when combined with gene editing tools like CRISPR-Cas9, enables targeted modification of specific genes for directed improvement of strain characteristics [209], facilitating the breeding of new varieties with enhanced yield, quality, and resilience. Marker-Assisted Selection (MAS) and Genomic Selection (GS) are already aiding the development of new edible mushroom lines exhibiting, for example, improved post-harvest quality [210]. The potential of using CRISPR to enhance cordycepin or ergothioneine production in *C. militaris* [33,211] and employing RNAi for gene expression regulation are prime examples of successful proof-of-concept studies, drawing inspiration from omics-assisted improvements in other crops [212]. Research institutes frequently report lab-scale successes in engineering strains with precisely edited genes for traits like increased metabolite production (e.g., 30–50% increase in cordycepin) or enhanced stress tolerance, though broad commercialization requires navigating regulatory hurdles, scale-up challenges, and market acceptance.

Optimizing Cultivation Processes and Active Substance Production: A deeper understanding of how environmental factors (e.g., light [121], temperature [117,118], humidity, CO_2_ concentration [183], substrate composition [162]) regulate growth and metabolism informs the design of more efficient and tailored cultivation protocols. Precise environmental control can be used to induce specific metabolic pathways, thereby enhancing the content of target products or improving overall quality attributes. For medicinal fungi, integrated omics approaches are crucial for elucidating the complex biosynthetic pathways of key active compounds, such as hericenones/erinacines in *Hericium* spp. [152], ganoderic acids in *Ganoderma* spp. [42,61], and cordycepin in *Cordyceps* spp. [78,157]. This knowledge provides the theoretical basis and molecular targets for rationally designing and constructing engineered strains for overproduction via metabolic engineering strategies [19,172]. Optimizing edible fungi as ‘cell factories’ for sustainable production of nutraceuticals or pharmaceutical intermediates is an area of active development, with producers likely adopting precise cultivation parameters based on omics data to improve yield and quality.

Enhancing Overall Industry Efficiency and Competitiveness: Fundamentally, deepening molecular research acts as a driving force for industrial upgrading [20]. Omics-driven technological innovations can not only improve the yield and quality of traditional edible mushroom products but also facilitate the development of novel, high-value products, including functional foods and drug lead compounds. A thorough exploration of the metabolic potential of diverse edible fungi, including the utilization of agricultural or industrial waste biomass as cultivation substrates [213], can open up new application domains. This promotes industrial diversification, enhances economic benefits, and bolsters the core competitiveness of the edible fungi sector [20]. Furthermore, research on mitochondrial genomics continues to contribute valuable insights for improving cultivation biotechnology [214].

### 7.2. Persistent Challenges and Untapped Potential

Expanding Scope and Depth: Current research predominantly focuses on a limited number of commercially significant species, leaving the vast metabolic potential of numerous wild edible and medicinal fungi largely untapped. Species like *Tuber* spp. (truffles), with their complex symbiotic lifestyles, and *Morchella* spp. (morels), despite increasing genomic and transcriptomic data, require more extensive proteomic investigation, particularly concerning cultivation-relevant traits like sclerotium formation or fruiting body development, and comparative proteomics across ecotypes. Similarly, *Sparassis* spp. (cauliflower mushrooms), valued for their medicinal properties, would benefit from deeper proteomics focusing on bioactive compound synthesis (e.g., β-glucan) [54] and stress responses [187]. Many regionally important, traditionally consumed wild mushrooms (e.g., from *Boletaceae*, *Russulaceae*) remain molecularly uncharacterized, representing a reservoir of untapped metabolic diversity. Even for well-studied commercial species like *L. edodes* or *P. ostreatus*, there is considerable scope for advanced proteomic studies, such as investigating the role of Post-Translational Modifications (PTMs) in metabolic regulation or performing comparative proteomics of diverse strains with distinct traits. Furthermore, a more profound understanding of the fine-tuned regulatory mechanisms (e.g., transcription factor networks, PTMs, non-coding RNA functions) governing metabolic networks in known species is essential. Finer spatio-temporal resolution in omics studies, potentially through single-cell omics, is also needed, though its current application in edible fungi is limited due to challenges in cell wall disruption and obtaining sufficient high-quality material from distinct fungal cell types within complex tissues.

Deepening Multi-Omics Integration and Predictive Modeling: While multi-omics integration is advancing, effectively integrating heterogeneous data, handling differences in data dimensionality and scale, accounting for biological time lags, and improving data completeness and accuracy remain core challenges (as detailed in Section 5.2). Current integration efforts often culminate in correlation analyses rather than the construction of truly predictive systems models, such as dynamic metabolic models or comprehensive gene regulatory networks. The application of Genome-Scale Metabolic Models (GSMMs) in edible fungi is still relatively nascent but is showing promise for species like *C. militaris* when combined with transcriptomic and exometabolomic data [207]. Developing integration strategies, algorithms, and bioinformatics tools specifically tailored for the unique characteristics of fungi (e.g., heterokaryosis, complex secondary metabolism) is imperative.

Addressing Bottlenecks in Metabolic Engineering: For effective metabolic engineering to enhance bioactive compound production, several bottlenecks need to be addressed: (i) incomplete understanding of complex regulatory networks (transcription factors, signaling, PTMs), making engineering outcomes unpredictable; (ii) ensuring adequate precursor supply and balancing metabolic flux, which requires redirecting central carbon metabolism without overburdening cell growth (GSMMs can aid here, but model construction and validation are demanding); (iii) considering subcellular compartmentalization of biosynthetic pathways and the targeting and transport of enzymes and intermediates; (iv) the lack of efficient and versatile genetic tools, including multiplex gene editing systems (e.g., CRISPRa/i), for many non-model edible fungi; (v) limited knowledge of transport and secretion mechanisms for extracellularly targeted products; and (vi) ensuring the genetic stability and scalability of engineered strains under industrial conditions.

Improving Translation from Basic Research to Industrial Application: Effectively converting fundamental omics knowledge into practical breeding strategies, optimized cultivation techniques, and novel product development represents a critical “last mile” challenge. This necessitates closer collaboration between research institutions and industry. While omics provides foundational knowledge and proof-of-concept for improvements (e.g., browning resistance in *L. edodes* via PPO gene insights or cordycepin enhancement in *C. militaris*), widespread commercial products explicitly and solely derived from very recent omics/CRISPR translation are still emerging. Patent activity and pilot-scale successes suggest active development, but the path to market is often long, involving scale-up, regulatory approvals, and market acceptance. This translational research is key to realizing the full industrial potential of edible fungi.

### 7.3. Concluding Outlook

Looking ahead, with ongoing advances in sequencing technologies (including long-read and single-molecule sequencing), the maturation of single-cell omics, enhanced bioinformatics capabilities, and the increasingly sophisticated application of gene editing tools like CRISPR-Cas9/Cas12a, research on edible fungi metabolism based on genomics, transcriptomics, proteomics, and their integration (as depicted conceptually in Figure 2) will enter a new era characterized by greater precision, systematic approaches, and improved efficiency. A profound understanding of how edible fungi construct and regulate their complex metabolic networks will not only satisfy fundamental scientific inquiry but also provide the crucial scientific foundation for the rational development and utilization of these valuable biological resources. This, in turn, will promote sustainable development of the edible fungi industry and contribute significantly to human health and well-being.

## Figures and Tables

**Table 1:** Applications of genomics in metabolic research of edible fungi.

Mushroom Species	Genome Research Type	Key Genes	Involved Metabolic Pathways	Significance	Ref.
*Agrocybe aegerita*	genome sequencing and hybrid assembly, gene annotation	genes for non-specific peroxidases, dye-decolorizing peroxidases, and Carbohydrate-active enzymes (CAZymes)	lignocellulose degradation	study of fruiting body development, biodegradation potential	[34]
*Cordyceps guangdongensis*	genome sequencing and annotation	CAZymes genes	biosynthesis, transport, and catabolism of secondary metabolites	provides basis for studying genetic and molecular mechanisms of fruiting body development	[35]
*Cordyceps militaris*	construction of genome-scale metabolic network (GSMM)	genes for biosynthesis of cordycepin and hydrolytic enzymes	biosynthesis of cordycepin, central carbon and amino acid metabolism	aids efficient production of high-value bioactive compounds with pharmaceutical potential	[36]
*Cordyceps militaris*	genome sequencing and annotation	genes for non-ribosomal peptide synthetases, type 1 polyketide synthases, and polyketide synthase-non-ribosomal peptide synthetase	biosynthesis of emercellamide and equisetin	further research and characterization of secondary metabolites produced by *C. militaris*	[37]
*Cordyceps militaris*	genome sequencing and annotation	genes for terpenoid cyclase, terpenoid synthase, fatty-acid synthase, and geranylgeranyl diphosphate synthase	biosynthesis of cordycepin	facilitates molecular studies on biology, fungicity, pathogenicity, medicinal compound production	[38]
*Cordyceps militaris*	genome bisulfite sequencing	genes for pyruvate metabolism, glycerophospholipid metabolism, DNA replication, and biosynthesis of N-glycan	pyruvate metabolism, glycerophospholipid metabolism, DNA replication, and N-glycan biosynthesis	helps understand potential mechanisms of strain degeneration in *C. militaris*	[39]
*Dictyophora indusiate*	secondary metabolite gene cluster analysis	genes for CYP450 family, and biosynthesis of terpene	biosynthesis of terpene	potential for medicinal compound production	[40]
*Flammulina velutipes* var. *lupinicola*	genome sequencing	lignocellulosic enzyme	lignocellulose degradation	understanding lignocellulose decomposition mechanisms	[41]
*Ganderma lucidum*	genome sequencing and annotation	genes for CAZymes and ligninolytic enzymes	biosynthesis of triterpene, wood degradation	aids bioengineering studies for active ingredient and bioenergy production	[42]
*Ganoderma leucocontextum*	genome sequencing and annotation, genome composition analysis	genes for CAZymes and biosynthesis of ergosterol	biosynthesis of sesquiterpenoid and triterpenoid	preliminary understanding of the biosynthesis of active secondary metabolites	[43]
*Ganoderma Lucidum*	genome resequencing and gene variation detection, annotation	genes for mycelial growth rate and synthesis of triterpene	synthesis of triterpene	reveals genetic mechanisms of *G. lucidum* mycelial growth and triterpene synthesis	[44]
*Gomphus purpuraceus*	genome sequencing and comparative genomics	genes for CAZymes and α-amylase family	starch/sucrose metabolism	studying biosynthesis of active compounds, understanding survival mechanisms and saprophytic ability	[30]
*Hericium erinaceus*	genome sequencing and annotation, gene identification	CAZymes genes	lignocellulose degradation	characterizes CAZymes and TFs in the genome, expands biological and genetic studies	[45]
*Lentinula edodes*	genome sequencing	genes for kinases and heat shock proteins	stress response	studying domestication process of Chinese *L. edodes* using population genomics	[46]
*Lentinula edodes*	genome sequence analysis	multicopper oxidase genes	expression of multicopper oxidases in mycelia, growing fruiting bodies, and fruiting bodies after harvest	suggests shared expression patterns and biological functions for laccases within the same group	[47]
*Lyophyllum decastes*	genome sequencing and annotation, CAZyme ID, mating type loci char.	laccase, quinone reductase, and CAZymes genes	lignocellulose degradation	understanding lignin degradation	[48]
*Morchella importuna*	genome sequencing, assembly and annotation, comparative analysis	CAZymes genes	starch degradation	better understanding of *Morchella* biology and evolution	[49]
*Morchella sextelata*	genome sequencing and annotation, comparative genomics	genes for ER repair protein 1 family, secondary metabolite, and CAZymes	posttranslational modification, protein turnover, amino acid transport and metabolism	discovery of bioactive compounds	[50]
*Ophiocordyceps sinensis* (Medicinal fungi)	genome sequencing and annotation	genes for type I polyketide synthases (PKSs), non-ribosomal peptide synthetases (NRPSs), and terpene synthase	biosynthesis of cordycepin, carbohydrate, amino acid, and energy metabolism	facilitates discovery and identification of novel secondary metabolite gene clusters, medicinal potential	[51]
*Oudemansiella raphanipes*	genome sequencing	CAZymes genes	biosynthesis of secondary metabolites, wood degradation	wood degradation and bioactive compound synthesis	[52]
*Pleurotus giganteus*	genome sequencing and assembly, comparative genomics	CAZymes genes	lignocellulose degradation, high-temperature adaptation	strong lignocellulolytic ability, development of molecular markers for ID and breeding	[53]
*Sparassis latifolia*	genome sequencing and comparison, SMGC analysis	genes encoding enzymes for carbohydrate and glycoconjugate metabolism, key SM biosynthesis enzymes	indole, terpene, and type I polyketide pathways	elucidates genetic basis of reported medicinal properties	[54]
*Stropharia rugosoannulata*	genome sequencing and comparative genomics	CAZymes genes	lignocellulose degradation	understanding lignocellulolytic ability	[55]
*Tricholoma matsutake*	comparative genomics and symbiotic adaptation	iron homeostasis genes	tryptophan metabolism	adaptation to symbiotic lifestyle and nutrient availability	[56]
*Volvariella volvacea*	genome sequencing and assembly, DEG identification	cellulase, hemicellulase, and pectinase genes	low temperature response, degradation of cellulose, hemicellulose, and pectin	provides evolutionary model for saprophytic nutrition and specific molecular mechanisms of cold sensitivity	[57]
*Volvariella volvacea*	genome sequencing and phylogenetic analysis	Flavin adenine dinucleotide (FAD)-binding proteins genes	energy transfer and utilization	understanding functional differences between homokaryons and heterokaryons	[58]
*Flammulina velutipes*	genome sequencing and key gene bioinformatics analysis	L-lysine biosynthesis genes	α-aminoadipate pathway	improving varieties for higher lysine content via genetic engineering	[59]

**Table 2:** Transcriptomic studies revealing gene expression in edible fungi.

Mushroom Species	Samples Collected Condition	Key DEGs	Key Pathways	Significance	Ref.
*Agaricus bisporus*	browning susceptible vs. resistant	polyphenol oxidase (PPO) genes	browning-related pathways	reveals browning mechanisms	[93]
*Agaricus bisporus*	six strains at different developmental stages	aminodeoxychorismate synthase, transcriptional enhancer factor genes, among others	fatty acid metabolism, biosynthesis of steroid and folate	reveals important DEGs in fruiting body development from different perspectives	[94]
*Agaricus bisporus*	caps at different post-harvest storage times	PPO genes	browning-related pathways	understanding PPO regulation of browning mechanism in *A. bisporus*	[95]
*Agaricus bisporus*	four developmental stages of fruiting body	DNA replication, base repair, RNA transport, ribosome etc.	amino acid, carbohydrate, nucleotide, lipid, and energy metabolism	reveals genes related to fruiting body growth and development	[96]
*Agaricus blazei*	different developmental stages of fruiting body	carbohydrate metabolism, fatty acid degradation, amino acid metabolism genes, among others	DNA replication, base excision repair, mismatch repair, RNA transport, and ribosome	understanding gene expression at different fruiting body developmental stages	[97]
*Agaricus blazei*	mycelia vs. primordia	response to stress genes	energy production	understanding pathways for polysaccharide and benzaldehyde biosynthesis, and fruiting body formation genes	[83]
*Auricularia auricula-judae*	three morphologically distinct fruiting bodies	peroxidase-like genes	starch and sucrose metabolism, MAPK signaling (yeast), biosynthesis of amino acid, secondary metabolite, and antibiotic	contributes sequence data to public databases, establishes relationships between major varieties	[98]
*Auricularia fibrillifera*	drought stress, rehydration	Tyrosinase and homogenesate1,2-dioxygenase genes, among others	resisting oxidative stress, osmotic adjustment	provides new potential targets for breeding and cultivation of drought-tolerant fungi	[88]
*Auricularia polytricha*	mycelia vs. mature fruiting bodies	tyrosinase genes	metabolism of amino acid, carbohydrate, energy, lipid, nucleotide	reveals candidate genes related to fruiting body formation	[99]
*Cordyceps cicadae*	three developmental stages	5′-nucleotidase and adenosine deaminase genes	cordycepin biosynthesis	enhancing understanding of biosynthesis of cordycepin and other characteristic secondary metabolites	[100]
*Cordyceps militari*	mycelia vs. fruiting bodies	genes for virulence and sexual development	energy metabolism, signaling pathways	suggests lncRNA expression regulates fungal virulence and sexual development by affecting gene expression	[101]
*Cordyceps militaris*	dark vs. light	*Cmtns* gene	biosynthesis of carotenoids	reveals carotenoid biosynthesis pathway and improving yield	[102]
*Cordyceps militaris*	dark vs. specific light	glyoxalase system genes	response to light/dark stimulation, biosynthesis of carotenoid and cordycepin	suggests transcriptional co-regulation plays a metabolic control role in light adaptation	[103]
*Cordyceps militaris*	six generations of artificial culture	genes for toxin biosynthesis, DNA methylation and chromatin remodeling, and energy metabolism	strain degeneration	reveals strain degeneration mechanism	[104]
*Cordyceps militaris*	albino mutant vs. normal strain	secondary metabolite backbone genes	response to light	understanding pigment biosynthesis pathway	[105]
*Cordyceps militaris*	submerged vs. liquid surface culture	adenylosuccinate synthetase and phosphoribosylaminoimidazole-succinocarboxamide (SAICAR) synthase genes	purine nucleotide metabolism	reveals cordycepin biosynthesis pathway and improving cordycepin production	[106]
*Cordyceps militaris*	different culture media	cell membrane, catalytic activity	carotenoid biosynthesis	understanding carotenoid biosynthesis pathway in *C. militaris*	[107]
*Cordyceps militaris*	culture with vs. without L-alanine addition	Zn2Cys6 type TFs	biological of energy production and amino acid transformation	supports improving cordycepin yield and strain breeding	[108]
*Cordyceps militaris*	different carbon sources	cordycepin biosynthesis genes	transcriptional regulation of central carbon metabolism	studying overall metabolic response of *C. militaris* for cordycepin production	[109]
*Cordyceps militaris*	xylose vs. other carbon sources	pentose and glucuronate interconversions	cordycepin biosynthesis	reveals response mechanism to xylose utilization	[110]
*Cordyceps militaris*	wild-type vs. wc-1 deficient strain	Cmwc-1(a homolog of the blue-light receptor gene white collar-1 (wc-1) in Neurospora crassa)	steroid biosynthesis	investigating role of blue light receptor gene (wc-1) in *C. militaris* fruiting and secondary metabolism	[32]
*Flammulina filiformis*	mycelia vs. primordia	genes for sugar transporter and unsaturated fatty acid synthesis	glycolysis, phospholipid and sphingolipid metabolism	reveals energy source transition during primordium formation	[111]
*Flammulina filiformis*	four developmental stages	heat stress-induced hsp70, hsp90, fes1 genes	heat stress response	studying gene function and improving mushroom heat tolerance	[112]
*Flammulina velutipes*	monokaryotic vs. dikaryotic mycelia	genes for transcription factors, protein kinases, and WD40 repeat-like proteins	synthesis of fatty acid, amino acid, and most saccharide	screens DEGs before and after mating in *F. velutipes*	[113]
*Flammulina velutipes*	mycelia vs. primordia	ribosome and DNA replication, among others	glycolysis, pentose phosphate pathway	reveals DEGs between F. velutipes dikaryotic mycelia, and primordia	[114]
*Ganoderma lucidum*	three continuous developmental stages	genes for carbohydrate metabolism, triterpenoid and ergosterol biosynthesis	ganoderic acids and ergosterol biosynthesis	reveals genes potentially involved in meiotic transcriptional control, metabolic pathways for energy supply, and biosynthesis of ganoderic acids and ergosterol	[115]
*Ganoderma lucidum*	addition of L-phenylalanine	genes for L-phenylalanine metabolism and cell wall mannoprotein	fungal polysaccharide production	provides effective strategy for high-yield, low-cost fungal polysaccharide production	[116]
*Ganoderma lucidum*	addition of PDE inhibitor or AC activator	genes for squalene synthase and lanosterol synthase	cAMP-induced apoptosis, ganoderic acids (GAs) biosynthesis	reveals cAMP signaling induces fungal apoptosis, and secondary metabolite production	[117]
*Ganoderma lucidum*	mycelia vs. fruiting bodies	FOLymes and CAZymes genes	terpene backbone biosynthesis pathway	provides comprehensive gene expression information	[118]
*Grifola frondosa*	mycelia	polysaccharide synthesis-related genes	polysaccharide synthesis	provides basis for studying polysaccharide metabolic pathways and related functional genes	[119]
*Lentinula edodes*	dikaryotic mycelia vs. mature fruiting bodies	peptidases and phosphotransferases genes	oxidative stress, starvation stress response	elucidating molecular mechanisms of mature fruiting body development and beneficial properties	[120]
*Lentinula edodes*	blue light vs. dark	morphogenesis and photoreception genes	blue light signaling pathway	identification of light-responsive genes	[121]
*Lentinula edodes*	monokaryotic mycelia with different laccase activity	cytochrome P450, glycoside hydrolase, and UDPG dehydrogenase genes	lignin degradation and carbohydrate metabolism	deeper understanding of physiological metabolism in high-laccase-yielding *L. edodes* strains	[122]
*Lentinula edodes*	dark vs. blue light	CAZymes genes	pentose and glucuronate conversion, starch and sucrose metabolism	aids functional studies of genes involved in developmental control	[123]
*Lentinula edodes*	different vegetative mycelial growth phenotypes (light exposure)	kinases, tyrosinase, glucanase, chitinase, and laccase genes	melanogenesis, cell wall degradation, signaling	analysis of expression patterns of light-induced browning-related genes	[124]
*Lyophyllum decastes*	five developmental stages	extracellular enzymes and TFs genes	signaling pathways	understanding fruiting body development	[125]
*Morchella importuna*	three mycelial growth stages	transketolase (tktA) and glucose-6-phosphate dehydrogenase (G6PDH) genes, among others	carbohydrate metabolism	elucidating mechanisms of mycelial growth	[126]
*Morchella importuna*	mycelia vs. young fruiting bodies	CAZymes, mitochondrial proteins, oxidoreductases, and HSPs genes	carbohydrate catabolism and energy metabolism	exploring fruiting body formation mechanisms in *M. importuna*	[127]
*Morchella importuna*	three mycelial growth stages	CAZymes genes	metabolism of carbohydrates, polysaccharides, hydrolases, caprolactam, β-galactosidase, and disaccharides	reveals carbohydrate catabolism mainly occurs during vegetative mycelial stage	[128]
*Morchella importuna*	mature fruiting bodies	genes for respiration, carbohydrate metabolism, tissue softening, and oxidative browning	molecular mechanism of post-harvest quality changes	provides theoretical basis for post-harvest quality change mechanisms and preservation technology	[129]
*Chinese cordyceps* (Medicinal fungi)	mycelia, sclerotia, and primordia	pheromone receptor and amino acid sensing genes,	DNA synthesis, cell division, MAPK pathway	reveals potential mechanisms of fruiting body initiation	[130]
*Ophiocordyceps sinensis* (Medicinal fungi)	mycelia vs. fruiting bodies	adenosine metabolism enzyme genes	cordycepin biosynthesis	provides data supporting elucidation of cordycepin biosynthesis mechanisms	[131]
*Ophiocordyceps sinensis* (Medicinal fungi)	fruiting bodies	pheromone receptors, G protein γ-subunit, G protein α/β subunits, and cordycepin synthesis enzymes genes	signaling, cordycepin biosynthesis	reveals genes associated with fruiting body development, and cordycepin biosynthesis	[87]
*Pleurotus eryngii*	different developmental stages	genes encoding enzymes involved in carbon and amino acid metabolism	carbon and amino acid metabolism	reveals gene expression changes during fruiting body growth and development	[132]
*Pleurotus eryngii*	transformed vs. wild-type strains	genes for mycelial growth and enzyme activity	MAPK signaling and inositol phosphate metabolism, among others	validates the functional role of GNAI in *P. eryngii* growth and development	[133]
*Pleurotus eryngii*	dark vs. blue light	CAZymes genes	carbon metabolism, glycolysis and biosynthesis of amino acids	understanding primordia response to blue light at developmental stage	[134]
*Pleurotus eryngii* subsp. *Tuoliensis*	cold stimulus, mycelia	genes for cell wall and membrane stability, Ca^2^⁺ signaling, MAPK pathway, and soluble sugar and protein biosynthesis	mycelia response to cold stimulation	understanding molecular mechanisms related to cold stimulus response	[135]
*Pleurotus ostreatus*	H_2_O_2_ regulation	genes in the respiratory chain	ATP synthesis	provides basis for breeding dark P. ostreatus strains and understanding pigmentation mechanism	[136]
*Pleurotus ostreatus*	heat stress	MYB gene family	mechanisms of fruiting body development and stress response	promotes further functional analysis of MYB genes	[137]
*Pleurotus pulmonarius*	mycelia vs. fruiting bodies	*PpFBD*1 (a gene that is highly expressed during the early stages of primordium formation)	synthesis of fungal cell walls	studying DEGs and their functions at different growth stages	[138]
*Pleurotus tuoliensis*	three developmental stages	morphogenesis genes	primary carbohydrate metabolism, cold stimulus and blue light response	indicates vegetative-to-reproductive transition as most active and critical period for gene expression changes	[139]
*Pleurotus tuoliensis*	immature vs. mature mycelia	genes encoding nucleoside diphosphate kinase, GH family proteins, and extracellular polygalacturonase	nucleotide synthesis and energy metabolism	understanding molecular mechanisms of mycelial maturation	[140]
*Pleurotus tuoliensis*	monokaryotic vs. dikaryotic strains	phenylalanine ammonium lyase and aryl-alcohol oxidase genes	catalytic activity and metabolic process	exploring fruiting body development genes, providing efficient markers for MAS	[141]
*Sparassis Primordia*	dark vs. light	genes associated with metabolism of vitamin B6 and selenocompound metabolism, among others	cysteine synthesis, vitamin B6 metabolism, glycine metabolism	establishes DE map for light-induced primordium formation	[142]
*Sparassis Primordia*	dark vs. light	glutathione S-transferase and HSP 9/12 genes, among others	secondary metabolite synthesis, starch and sucrose metabolism, glutathione metabolism	analysis of molecular mechanisms of light response	[143]
*Stropharia rugosoannulata*	cold stress	carbohydrate enzyme genes	metabolism of carbohydrate, lipid, and xenobiotic	reveals cold resistance mechanisms	[144]
*Tremella fuciformis*	mycelia	L-iditol 2-dehydrogenase and butanol dehydrogenase genes	polysaccharide metabolic pathways	provides data support for studying polysaccharide and other product biosynthesis pathways and mechanisms	[145]
*Volvariella volvacea*	different commercial strains	heat shock proteins genes	stress response	identifying candidate genes involved in rapid growth requirements	[146]
*Wolfiporia cocos*	mycelia vs. sclerotia	diphosphomevalonate decarboxylase and farnesyl diphosphate synthase genes, among others	biosynthesis of triterpenoids	reveals genes related to triterpenoid biosynthesis	[147]

**Table 3:** Proteomic analysis of metabolic pathways and protein networks in edible fungi.

Mushroom Species	Proteomic Research	Key Proteins	Key Pathways	Significance	Ref.
*Auricularia auricula-judae*	effect of freezing on melanin accumulation mechanism	glycolysis/gluconeogenesis proteins	tyrosine metabolism, ribosome and arginine biosynthesis	provides information on the mechanism of freezing treatment effects on color quality	[158]
*Hypsizygus marmoreus*	protein changes from “scratching” to primordia	oxidoreductases, peptidases, hydrolases	catabolic and carbohydrate-related processes	understanding developmental changes preceding primordia formation	[159]
*Hypsizygus marmoreus*	protein expression during mycelial growth	proteins involved in carbohydrate metabolism, catabolic processes, oxidoreductase activity	carbohydrate metabolism, catabolic processes, oxidoreductase activity	elucidating protein changes during development	[160]
*Hypsizygus marmoreus*	heat stress response	SOD and peroxidase, trehalose synthase, heat shock proteins (HSPs)	expression of catalase	understanding molecular mechanisms of heat stress response	[161]
*Lentinula edodes*	substrate effect on protein expression	proteins involved in carbohydrate and oxidoreductase activity pathways	carbohydrate and oxidoreductase activity pathways	cultivation effects on nutritional quality	[162]
*Lyophyllum decastes*	lignin degradation	laccase, quinone reductase	lignocellulose degradation	confirms lignin degradation mechanism	[48]
*Morchella importuna*	proteome analysis of vegetative vs. sexual stages	lectin proteins	carbohydrate and amino acid metabolic pathways	understanding nutrient metabolism and fruiting body development	[163]
*Ophiocordyceps sinensis* (Medicinal fungi)	Proteome analysis of three key dev. stages	ROS-related proteins, proteins involved in carbon transport and metabolism, response to oxidative stress, antioxidant activity and translation	carbon transport and mechanism, response to oxidative stress, antioxidative activity	understanding biological traits of fruiting body development and high-altitude adaptation	[164]
*Schizophyllum commun*	trehalose biosynthesis pathway	trehalose synthase, trehalose phosphatase, trehalose phosphorylase	growth and development of mycelium, trehalose biosynthesis	potential for industrial trehalose production	[165]
*Stropharia rugosoannulata*	tissue-specific protein expression	proteins for carbon metabolism, energy production, stress response	fatty acid synthesis, mRNA splicing	understanding tissue-specific metabolism	[144]
*Tremella fuciformis*	mechanism and proteomics of conidia–mycelia transition	proteins involved in biosynthesis, DNA replication, and DNA damage repair, among others	MAPK signaling pathway	provides basis for studying dimorphism formation mechanisms	[166]
*Volvariella volvacea*	post-harvest autolysis mechanism	proteins involved in fatty acid metabolism and RNA transport	RNA transport, biosynthesis of fatty acid and amino acid	provides reference for further studies on senescence mechanisms	[167]

**Table 4:** Integrative omics studies providing insights into the metabolism of edible fungi.

Mushroom Species	Integrated Omics Technologies	Research Question/Focus	Key Insights Obtained	Ref.
*Agaricus blazei*	genomics, transcriptomics	gene function, DEG between mycelia and primordia stages	understanding pathways for polysaccharide and benzaldehyde biosynthesis, fruiting body formation related genes	[83]
*Agrocybe aegerita*	transcriptomics, proteomics	mycelia vs. fruiting bodies	provides clues for nutritional, pharmaceutical, and industrial applications	[157]
*Auricularia cornea*	genomics, transcriptomics, metabolomics	high-quality genome assembly and multi-omics analysis of pigment synthesis pathway	revealed genetic blueprint, evolutionary history, and pigment synthesis mechanism	[190]
*Cordyceps cicadae*	genomics, transcriptomics, metabolomics	asexual fruiting body formation characteristics and secondary metabolism	understanding fungal genetics and promoting medicinal research	[191]
*Cordyceps kyushuensis*	transcriptomics, proteomics	single gene cluster for cordycepin and pentostatin biosynthesis	for improving cordycepin yield and finding more functional proteins	[92]
*Cordyceps militaris*	genomics, transcriptomics	genome, transcriptome, and methylome of a new strain	provides basis for fungal molecular biology	[192]
*Cordyceps militaris*	transcriptomics, proteomics	gene expression differences between mycelia and fruiting bodies	facilitates systematic molecular studies of developmental stages	[193]
*Dictyophora indusiate*	genomics, transcriptomics, metabolomics	fruiting body differentiation	importance of tryptophan metabolism	[40]
*Flammulina filiformis*	genomics, comp. transcriptomics	expression of biosynthetic gene clusters (BGCs) in wild/cultivated strains and dev. stages	elucidating regulation of secondary metabolites	[194]
*F. filiformis*	genomics, transcriptomics	role of cytochrome c peroxidase gene (*FfCcP*) in dev. and stress response	upregulation of *FfCcP* might facilitate stipe elongation	[195]
*F. velutiper*, *G. Lucidum*, *H. erinaceus*, *H. marmoreus*, *L. edodes*, *P. eryngii*, *P. geesteranus*, *V. volvacea*	comp. metabolomics, proteomics	ameliorative effect of melatonin (MT) on Cd-induced oxidative stress	confirmed widespread presence of MT in edible fungi and its important role in physiological regulation	[196]
*Flammulina velutipes*	transcriptomics, metabolomics	regulation of ergosterol biosynthesis	understanding ergosterol biosynthesis regulation during fruiting and metabolite–gene relationships	[197]
*Ganoderma lucidum*	genomics, transcriptomics	RNA editing	elucidating role of transcriptional plasticity in dev., environmental adaptation, and SM pathway regulation	[198]
*Ganoderma lucidum*	genomics, transcriptomics	lignocellulolytic enzymes	understanding changes in lignocellulose degradation ability during *G. lucidum* growth	[199]
*Ganoderma lucidum*	genomics, transcriptomics	key genes (Cyt P450s, transporters, regulators), triterpenoid biosynthesis	potential model system for studying SM pathways and regulation in medicinal fungi	[61]
*Lentinula edodes*	proteomics, metabolomics	substrate effect on metabolism and nutrition	linking substrate to protein/metabolite changes	[162]
*Morchella importuna*	genomics, transcriptomics	response mechanisms at different stages post-*Paecilomyces* infection	fungus–fungus interactions involving edible fungi	[174]
*Morchella sextelata*	transcriptomics, proteomics	heat shock response, development, pathogen infection	heat shock adaptation network; role of sugar metabolism; defense mechanisms against pathogens	[200]
*P. eryngii var. eryngii*, *Pleurotus tuoliensis*	genomics, transcriptomics	DNA methylation and gene expression during three major developmental transitions	epigenetic and transcriptional regulatory mechanisms supporting gene expression during development	[201]
*Phlebopus portentosus*	genomics, transcriptomics	lignocellulose degradation system and fruiting body development	understanding key pathways and hub genes involved in development	[202]
*Pleurotus giganteus*	transcriptomics, proteomics, nutritional analysis	substrate and mRNA transport within fruiting body	complex interactions between gene expression, protein synthesis, and nutrient allocation	[184]
*Pleurotus ostreatus*	genomics, transcriptomics, proteomics	secreted proteins and environmental interaction	secreted protein transcription depends more on strain genotype than environment	[178]
*Pleurotus ostreatus*	genomics, proteomics, metabolomics	secretome of strains on different substrates	identifying enzymes overproduced in lignocellulose cultures	[203]
*Volvariella volvacea*	genomics, transcriptomics, proteomics	low-temperature autolysis	cold stress might trigger VvAgo1-mediated RNAi to promote low-temperature autolysis	[170]
*Volvariella volvacea*	genomics, transcriptomics	composition and expression of CAZyme-encoding genes	identified CAZymes, showed differential expression between heterokaryon and homokaryon	[81]

## References

[1] Boa E. (2007). Wild Edible Fungi: A Global Overview of Their Use and Importance to People.

[2] Dai Y.-C., Yang Z.-L., Cui B.-K., Yu C.-J., Zhou L.-W. (2009). Species Diversity and Utilization of Medicinal Mushrooms and Fungi in China (Review). Int. J. Med. Mushrooms.

[3] Hu X.-R., Xiong H.-K., Xue W.T. (2017). Research progress on the composition and functional activity of truffles. Sci. Technol. Food Ind..

[4] Ma W.W., Pan S.-H. (2008). Research Advancement of *Cordyceps militaris*. Asia-Pac. Tradit. Med..

[5] Ai Z., Qian Z.-M., Li W.J., Yang F.Q., Chen S.L., Li E.-W. (2016). Recent advances in the analysis of nucleosides in Chinese cordyceps. Mycosystema.

[6] Yan W.-J., Lee T.-H., Tang F.-Y., Zheng B.-S., Jiang Z.-D. (2009). Extraction and Contents Determination of Polysaccharide in *Cordyceps guangdongensis*. J. South China Agric. Univ..

[7] Qian Z.-M., Li W.-Q., Sun M.-T., Liu X.-Z., Li E.-W., Li W.-J. (2016). Analysis of chemical compounds in Chinese cordyceps. Mycosystema.

[8] Guo T., Hou C.-L., Wei L., Sun J., Fan L. (2010). Antioxidant activities of extracts and sub-fractions from Tuber indicum. Mycosystema.

[9] Wang L.-M., Du X.-F., Li X.-J., Li S.-Y. (2009). Study on inhibition of proliferation and induction of apoptosis in BGC-823 cell by cordycep militaris. Chin. J. Microecol..

[10] Wu Z.-W., Wang Y.-B., Zhao X.-F., Dou Y.-P. (2008). Antibacterial Activity of Fermentation Broth by *Cordyceps sinensis* and *Cordyceps militaris*. J. Microbiol..

[11] Shi Q.-Y., Shao W.-P. (2003). Determination of nutritive components of eight edible fungi. J. Gansu Agric. Univ..

[12] Yang G. (2000). Nutritional Component of Einght Species of Fungi of Basidiomycetes. J. Food Sci. Biotechnol..

[13] Xiao X.-N., Yuan Y., Liao X., Wang L.-Y., Shi F., Ming J. (2016). Research advance on antioxidant active composition and antioxidant mechanisms of edible fungi. Food Mach..

[14] Chen K.-X., Wang W.-L., Liu J., Zhang F.-C., Zheng X.-F. (2015). Advance in Antitumor Mechanism of Bioactive Compounds in Edible Mushrooms. Biotechnol. Bull..

[15] Niu J., Wang A., Shi C., Zhuo L.-J., Xie Y.-Z., Li W.-Z., Hu H.-P. (2020). Advance Research on Hypoglycemic Effect of Edible Fungi in China. Edible Fungi China.

[16] Arshadi N., Nouri H., Moghimi H. (2023). Increasing the production of the bioactive compounds in medicinal mushrooms: An omics perspective. Microb. Cell Factories.

[17] Pang Y.-J., Lyu J., Yu C.-Q., Sun D.-J.Y., Li L.-M. (2021). A multi-omics approach to investigate the etiology of non-communicable diseases:recent advance and applications. Chin. J. Epidemiol..

[18] Cheng S., Zeng Y.-L., Li H., Wang Y.-J. (2022). Multiomics Studies Facilitate Stroke Drug Development. Chin. J. Stroke.

[19] Dong L.-Q., Gao Q. (2022). Multi-omics molecular subgrouping of hepatocellular carcinoma and its application in precision diagnosis and treatment. J. Clin. Hepatol..

[20] Cao L., Zhang Q., Miao R., Lin J., Feng R., Ni Y., Li W., Yang D., Zhao X. (2023). Application of omics technology in the research on edible fungi. Curr. Res. Food Sci..

[21] Velculescu V.E., Zhang L., Zhou W., Vogelstein J., Basrai M.A., Bassett D.E., Hieter P., Vogelstein B., Kinzler K.W. (1997). Characterization of the Yeast Transcriptome. Cell.

[22] Wang Y.-C., Dang Y., Li X.-Y., Wang X.-L. (2010). Proteomics and the development of Proteomics Techniques. Lett. Biotechnol..

[23] Al-Obaidi J.R. (2016). Proteomics of edible mushrooms: A mini-review. Electrophoresis.

[24] Yuan X.-H., Fu Y.-P., Xiao S.-J., Li C.-T., Wang D., Li Y. (2021). Research progress on mushroom phenotyping. Mycosystema.

[25] Agrawal G.K., Sarkar A., Righetti P.G., Pedreschi R., Carpentier S., Wang T., Barkla B.J., Kohli A., Ndimba B.K., Bykova N.V. (2013). A decade of plant proteomics and mass spectrometry: Translation of technical advancements to food security and safety issues. Mass. Spectrom. Rev..

[26] Cha L., Zhao R.-H., Yu C.-X., Zhao Y., Chen H., Zhang J.-J., Chen M.-J. (2017). Proteomics and Its Advance in Edible Fungi. Mol. Plant Breed..

[27] Yu Y., Zhang Y.-L. (2021). Research progress of omics analysis techniques in edible fungi. China Food Saf. Mag..

[28] Cao Z.-J., Hu B., Xu Z.-X., Zheng S.-Y., Wang C.-X. (2022). Research Progress on Transcriptome of Edible Fungi Under Adversity Stress. North. Hortic..

[29] Yang R., Li Y., Song X., Tang L., Li C., Tan Q., Bao D. (2017). The complete mitochondrial genome of the widely cultivated edible fungus *Lentinula edodes*. Mitochondrial DNA Part B.

[30] Geng Y., Zhang S., Yang N., Qin L. (2022). Whole-Genome Sequencing and Comparative Genomics Analysis of the Wild Edible Mushroom (*Gomphus purpuraceus*) Provide Insights into Its Potential Food Application and Artificial Domestication. Genes.

[31] Yin L.-W., Chi Y.-J. (2015). Cloning and Bioinformatics Analysis of Mn P1 cDNA Gene from Hericium erinaceum. Sci. Silvae Sin..

[32] Yang T., Guo M., Yang H., Guo S., Dong C. (2016). The blue-light receptor CmWC-1 mediates fruit body development and secondary metabolism in *Cordyceps militaris*. Appl. Microbiol. Biotechnol..

[33] Katrolia P., Liu X., Zhao Y., Kopparapu N.K., Zheng X. (2020). Gene cloning, expression and homology modeling of first fibrinolytic enzyme from mushroom (*Cordyceps militaris*). Int. J. Biol. Macromol..

[34] Gupta D.K., Rühl M., Mishra B., Kleofas V., Hofrichter M., Herzog R., Pecyna M.J., Sharma R., Kellner H., Hennicke F. (2018). The genome sequence of the commercially cultivated mushroom Agrocybe aegerita reveals a conserved repertoire of fruiting-related genes and a versatile suite of biopolymer-degrading enzymes. BMC Genom..

[35] Zhang C., Deng W., Yan W., Li T. (2018). Whole Genome Sequence of an Edible and Potential Medicinal Fungus, *Cordyceps guangdongensis*. G3 Genes|Genomes|Genet..

[36] Vongsangnak W., Raethong N., Mujchariyakul W., Nguyen N.N., Leong H.W., Laoteng K. (2017). Genome-scale metabolic network of *Cordyceps militaris* useful for comparative analysis of entomopathogenic fungi. Gene.

[37] Kramer G.J., Nodwell J.R. (2017). Chromosome level assembly and secondary metabolite potential of the parasitic fungus *Cordyceps militaris*. BMC Genom..

[38] Zheng P., Xia Y., Xiao G., Xiong C., Hu X., Zhang S., Zheng H., Huang Y., Zhou Y., Wang S. (2011). Genome sequence of the insect pathogenic fungus *Cordyceps militaris*, a valued traditional chinese medicine. Genome Biol..

[39] Xin X., Yin J., Zhang B., Li Z., Zhao S., Gui Z. (2019). Genome-wide analysis of DNA methylation in subcultured *Cordyceps militaris*. Arch. Microbiol..

[40] Duan M., Long S., Wu X., Feng B., Qin S., Li Y., Li X., Li C., Zhao C., Wang L. (2023). Genome, transcriptome, and metabolome analyses provide new insights into the resource development in an edible fungus *Dictyophora indusiata*. Front. Microbiol..

[41] Yu H.W., Im J.H., Kong W.S., Park Y.J. (2021). Comparative Analysis of Carbohydrate Active Enzymes in the *Flammulina velutipes* var. lupinicola Genome. Microorganisms.

[42] Liu D., Gong J., Dai W., Kang X., Huang Z., Zhang H.-M., Liu W., Liu L., Ma J., Xia Z. (2012). The Genome of Ganderma lucidum Provide Insights into Triterpense Biosynthesis and Wood Degradation. PLoS ONE.

[43] Liu Y., Huang L., Hu H., Cai M., Liang X., Li X., Zhang Z., Xie Y., Xiao C., Chen S. (2021). Whole-genome assembly of *Ganoderma leucocontextum* (Ganodermataceae, Fungi) discovered from the Tibetan Plateau of China. G3 Genes|Genomes|Genet..

[44] Zhu F.-L., Xu X.-L., Chen T.-Q., Shi L.-C., Miao X.-Q., Lan J. (2019). The Analysis of Whole-genome Resequencing of *Ganoderma lucidum* Originated from South Korea. Mod. Tradit. Chin. Med. Mater. Medica-World Sci. Technol..

[45] Gong W., Wang Y., Xie C., Zhou Y., Zhu Z., Peng Y. (2020). Whole genome sequence of an edible and medicinal mushroom, *Hericium erinaceus* (Basidiomycota, Fungi). Genomics.

[46] Xiao Y., Cheng X., Liu J., Li C., Nong W., Bian Y., Cheung M.K., Kwan H.S. (2016). Population genomic analysis uncovers environmental stress-driven selection and adaptation of *Lentinula edodes* population in China. Sci. Rep..

[47] Sakamoto Y., Nakade K., Yoshida K., Natsume S., Miyazaki K., Sato S., van Peer A.F., Konno N. (2015). Grouping of multicopper oxidases in *Lentinula edodes* by sequence similarities and expression patterns. AMB Express.

[48] Xu L., Yang W., Qiu T., Gao X., Zhang H., Zhang S., Cui H., Guo L., Yu H., Yu H. (2023). Complete genome sequences and comparative secretomic analysis for the industrially cultivated edible mushroom Lyophyllum decastes reveals insights on evolution and lignocellulose degradation potential. Front. Microbiol..

[49] Liu W., Chen L., Cai Y., Zhang Q., Bian Y. (2018). Opposite Polarity Monospore Genome De Novo Sequencing and Comparative Analysis Reveal the Possible Heterothallic Life Cycle of Morchella importuna. Int. J. Mol. Sci..

[50] Mei H., Qingshan W., Baiyintala W. (2019). The whole-genome sequence analysis of Morchella sextelata. Sci. Rep..

[51] Shu R., Zhang J., Meng Q., Zhang H., Zhou G., Li M., Wu P., Zhao Y., Chen C., Qin Q. (2020). A New High-Quality Draft Genome Assembly of the Chinese Cordyceps *Ophiocordyceps sinensis*. Genome Biol. Evol..

[52] Zhu L., Gao X., Zhang M., Hu C., Yang W., Guo L., Yang S., Yu H., Yu H. (2023). Whole Genome Sequence of an Edible Mushroom *Oudemansiella raphanipes* (Changgengu). J. Fungi.

[53] Yu H., Zhang M., Sun Y., Li Q., Liu J., Song C., Shang X., Tan Q., Zhang L., Yu H. (2022). Whole-genome sequence of a high-temperature edible mushroom *Pleurotus giganteus* (zhudugu). Front. Microbiol..

[54] Xiao D., Ma L., Yang C., Ying Z., Jiang X., Lin Y.Q. (2018). De Novo Sequencing of a Sparassis latifolia Genome and Its Associated Comparative Analyses. Can. J. Infect. Dis. Med. Microbiol..

[55] Li S., Zhao S., Hu C., Mao C., Guo L., Yu H., Yu H. (2022). Whole Genome Sequence of an Edible Mushroom *Stropharia rugosoannulata* (Daqiugaigu). J. Fungi.

[56] Kim J.H., Bae E.-K., Hue Y., Choi B., Kang M.-J., Park E.-J., Kim K.-T. (2024). Comparative Genomics Reveals Species-Specific Genes and Symbiotic Adaptations in Tricholoma matsutake. J. Fungi.

[57] Bao D., Gong M., Zheng H., Chen M., Zhang L., Wang H., Jiang J., Wu L., Zhu Y., Zhu G. (2013). Sequencing and Comparative Analysis of the Straw Mushroom (*Volvariella volvacea*) Genome. PLoS ONE.

[58] Meng L., Yan J., Xie B., Li Y., Chen B., Liu S., Li D., Yang Z., Zeng X., Deng Y. (2013). Genes encoding FAD-binding proteins in *Volvariella volvacea* exhibit differential expression in homokaryons and heterokaryons. Microbiol. Res..

[59] Liu J.-Y., Wang R.-J., Zhang D., Shang X.-D., Tan Q. (2016). Analysis of genes related to lysine biosynthesis based on whole genome of *Flammulina velutipes*. Microbiol. China.

[60] Kurata A., Fukuta Y., Mori M., Kishimoto N., Shirasaka N. (2016). Draft Genome Sequence of the Basidiomycetous Fungus *Flammulina velutipes* TR19. Genome Announc..

[61] Chen S., Xu J., Liu C., Zhu Y., Nelson D.R., Zhou S., Li C., Wang L., Guo X., Sun Y. (2012). Genome sequence of the model medicinal mushroom *Ganoderma lucidum*. Nat. Commun..

[62] Li J., Zhang J., Chen H., Chen X., Lan J., Liu C. (2013). Complete Mitochondrial Genome of the Medicinal Mushroom *Ganoderma lucidum*. PLoS ONE.

[63] Yang R., Li Y., Li C., Xu J., Bao D. (2016). The complete mitochondrial genome of the Basidiomycete edible fungus *Pleurotus eryngii*. Mitochondrial DNA Part B.

[64] Wang Y., Zeng F., Hon C.C., Zhang Y., Leung F.C.C. (2008). The mitochondrial genome of the Basidiomycete fungus *Pleurotus ostreatus* (oyster mushroom). FEMS Microbiol. Lett..

[65] Wan J., Li Y., Wang H., Tang L., Li Z., Zhou C., Tan Q., Bao D., Yang R. (2018). Three complete mitochondrial genomes of straw-rotting edible fungus *Volvariella volvacea* using next generation sequencing. Mitochondrial DNA Part B.

[66] Yu F., Zhang Y., Song J., Liang J. (2019). The complete mitochondrial genome of a wild edible mushroom, Russula griseocarnosa. Mitochondrial DNA Part B.

[67] Wang S.-R., Zhang J.-P., He Y.-R., Chang M.-C., Meng J.-L. (2021). Characterization of the complete mitochondrial genome of Coprinellus micaceus, a wild saprobic mushroom in China. Mitochondrial DNA Part B.

[68] Song Y., Wan J., Shang J.-J., Feng Z., Jin Y., Li H., Guo T., Wu Y.-Y., Bao D.-P., Zhang M. (2022). The complete mitochondrial genome of the edible mushroom Grifola frondosa. Mitochondrial DNA Part B.

[69] Xu W.-N., Huang R.-M., Liu Y.-Y., Tong Z.-J., Han X., Xie L.-Y., Xie B.-G. (2018). Genome sequencing and assembly strategy analyses of *Flammulina filiformis*. Mycosystema.

[70] Xu W.-N., Huang R.-M., Liu Y.-Y., Xie L.-Y., Jiang Y.-J., Xie B.-G. (2018). Sequence analyses of the rDNA structure of *Flammulina filiformis*. Mycosystema.

[71] Bao D.-P., Zhao G.-P., Tan Q., Wang S.-Y., Chen M.-J., Zheng H.-J., Zhang J.-S., Zhu Y.-Q., Wang H., Kang H. (2010). Draft Sequence of the *Volvariella volvacea* Genome. Acta Edulis Fungi.

[72] Yue G.-D., Gao Q., Luo L.-H., Wang J.-Y., Xu J.-H., Yin Y. (2012). The Application of High-throughput Sequencing Technology in Plant and Animal Research. Sci. Sin..

[73] Zhao Y., Liu Y., Chen X., Xiao J. (2023). Genome resequencing and transcriptome analysis reveal the molecular mechanism of albinism in *Cordyceps militaris*. Front. Microbiol..

[74] Wang C., Zhong H., Long X., Xu R., Gong Y., Bian Y., Zhou Y. (2023). The T-DNA integration characteristics of monokaryotic mutant library in *Lentinula edodes*. Sci. Hortic..

[75] Gong W., Xie C., Zhou Y., Zhu Z., Wang Y., Peng Y. (2020). A Resequencing-Based Ultradense Genetic Map of *Hericium erinaceus* for Anchoring Genome Sequences and Identifying Genetic Loci Associated with Monokaryon Growth. Front. Microbiol..

[76] Yu H., Zhang L., Shang X., Peng B., Li Y., Xiao S., Tan Q., Fu Y. (2022). Chromosomal genome and population genetic analyses to reveal genetic architecture, breeding history and genes related to cadmium accumulation in *Lentinula edodes*. BMC Genom..

[77] Liu Y.-Y., Xie L.-Y., Li Y.-N., Ma X.-B., Yang H., Wang M., Xie B.-G. (2020). Identification of exogenous DNA insertion site of transformants in *Flammulina filiformis* using matrix design. Mycosystema.

[78] Chang J.-L., Guo Y.-S., Yang D.-G., Li L.-W. (2008). The Research of Overview in Plant for Functional Genomics Techniques. J. Maize Sci..

[79] Wang J.-X., Sun Y., Xu P.-L., Yi Z.-B., Du J.-Z., Sun D.-Q. (2004). Research Progress in Functional Plant Genomics. Biotechnol. Bull..

[80] Gu F.-Y., Chen Z.-Y., Shi J.-J., Qian S.-J. (2008). Advances in Cellulase and Its Development Tendency. J. Microbiol..

[81] Chen B., Gui F., Xie B., Deng Y., Sun X., Lin M., Tao Y., Li S. (2013). Composition and Expression of Genes Encoding Carbohydrate-Active Enzymes in the Straw-Degrading Mushroom *Volvariella volvacea*. PLoS ONE.

[82] Yang F., Xu B., Li J., Huang Z. (2012). Transcriptome analysis of Termitomyces albuminosus reveals the biodegradation of lignocellulose. Acta Microbiol. Sin..

[83] Lu Y.-P., Liao J.-H., Guo Z.-J., Cai Z.-X., Chen M.-Y. (2020). Genome Survey and Transcriptome Analysis on Mycelia and Primordia of *Agaricus blazei*. BioMed Res. Int..

[84] Mitsis T., Efthimiadou A., Bacopoulou F., Vlachakis D., Eliopoulos E. (2020). Transcription factors and evolution: An integral part of gene expression (Review). World Acad. Sci. J..

[85] Hou Z., Chen Q., Zhao M., Huang C., Wu X. (2020). Genome-wide characterization of the Zn(II)2Cys6 zinc cluster-encoding gene family in *Pleurotus ostreatus* and expression analyses of this family during developmental stages and under heat stress. PeerJ.

[86] Langer I., Jeandriens J., Couvineau A., Sanmukh S., Latek D. (2022). Signal Transduction by VIP and PACAP Receptors. Biomedicines.

[87] Xiang L., Li Y., Zhu Y., Luo H., Li C., Xu X., Sun C., Song J., Shi L., He L. (2014). Transcriptome analysis of the *Ophiocordyceps sinensis* fruiting body reveals putative genes involved in fruiting body development and cordycepin biosynthesis. Genomics.

[88] Wang Y., Yang Z., Shi L., Yang R., Guo H., Zhang S., Geng G. (2022). Transcriptome analysis of Auricularia fibrillifera fruit-body responses to drought stress and rehydration. BMC Genom..

[89] Ferstl R., Akdis C.A., O’Mahony L. (2012). Histamine regulation of innate and adaptive immunity. Front. Biosci..

[90] Xu L.-M., Hinsinger D.D., Jiang G.-F. (2018). The complete mitochondrial genome of the Basidiomycete fungus Pleurotus cornucopiae (Paulet) Rolland. Mitochondrial DNA Part B.

[91] Bashir K.M.I., Rheu K.M., Kim M.-S., Cho M.-G. (2020). The complete mitochondrial genome of an edible mushroom, Sparassis crispa. Mitochondrial DNA Part B.

[92] Zhao X., Zhang G., Li C., Ling J. (2019). Cordycepin and pentostatin biosynthesis gene identified through transcriptome and proteomics analysis of Cordyceps kyushuensis Kob. Microbiol. Res..

[93] Cai Z.X., Chen M.Y., Lu Y.P., Guo Z.J., Zeng Z.H., Liao J.H., Zeng H. (2022). Metabolomics and transcriptomics unravel the mechanism of browning resistance in *Agaricus bisporus*. PLoS ONE.

[94] Shi X.-K., Lu Y.-P., Cai Z.-X., Guo Z.-J., Chen M.-Y., Liao J.-H. (2019). Transcriptome Sequencing on SixAgaricus bisporusStrains at Four Developmental Stages. Fujian J. Agric. Sci..

[95] Peng B., Li B.-J., Guan W.-Q., Lin Q. (2019). Transcriptome Sequencing of *Agaricus bisporus* and Mining of Genes Involved in Browning. Food Sci..

[96] Shi X.-K., Cai Z.-X., Guo Z.-J., Lu Y.-P., Chen M.-Y., Liao J.-H., Wang Z.-S. (2018). Transcriptome Sequencing on Fruiting Body of *Agaricus bisporus* in Developing Stages. Fujian J. Agric. Sci..

[97] Lu Y.-P., Guo Z.-J., Cai Z.-X., Chen M.-Y., Liao J.-H. (2019). Transcriptome analysis of *Agaricus blazei* fruiting bodies at different developmental stages. Mycosystema.

[98] Zhao Y., Wang L., Zhang D., Li R., Cheng T., Zhang Y., Liu X., Wong G., Tang Y., Wang H. (2019). Comparative transcriptome analysis reveals relationship of three major domesticated varieties of Auricularia auricula-judae. Sci. Rep..

[99] Zhou Y., Chen L., Fan X., Bian Y. (2014). De Novo Assembly of Auricularia polytricha Transcriptome Using Illumina Sequencing for Gene Discovery and SSR Marker Identification. PLoS ONE.

[100] Liu T., Liu Z., Yao X., Huang Y., Qu Q., Shi X., Zhang H., Shi X. (2018). Identification of cordycepin biosynthesis-related genes through de novo transcriptome assembly and analysis in Cordyceps cicadae. R. Soc. Open. Sci..

[101] Wang Y., Shao Y., Zhu Y., Wang K., Ma B., Zhou Q., Chen A., Chen H. (2019). XRN1-associated long non-coding RNAs may contribute to fungal virulence and sexual development in entomopathogenic fungus *Cordyceps militaris*. Pest. Manag. Sci..

[102] Lou H.W., Zhao Y., Tang H.B., Ye Z.W., Wei T., Lin J.F., Guo L.Q. (2019). Transcriptome Analysis of *Cordyceps militaris* Reveals Genes Associated With Carotenoid Synthesis and Identification of the Function of the Cmtns Gene. Front. Microbiol..

[103] Thananusak R., Laoteng K., Raethong N., Zhang Y., Vongsangnak W. (2020). Metabolic Responses of Carotenoid and Cordycepin Biosynthetic Pathways in *Cordyceps militaris* under Light-Programming Exposure through Genome-Wide Transcriptional Analysis. Biology.

[104] Yin J., Xin X., Weng Y., Gui Z. (2017). Transcriptome-wide analysis reveals the progress of *Cordyceps militaris* subculture degeneration. PLoS ONE.

[105] Wang F., Liu Q., Zhang J., Liu K., Li K., Liu G., Dong C. (2018). Comparative Transcriptome Analysis Between a Spontaneous Albino Mutant and Its Sibling Strain of *Cordyceps militaris* in Response to Light Stress. Front. Microbiol..

[106] Suparmin A., Kato T., Dohra H., Park E.Y. (2017). Insight into cordycepin biosynthesis of *Cordyceps militaris*: Comparison between a liquid surface culture and a submerged culture through transcriptomic analysis. PLoS ONE.

[107] Lou H.-W., Zhao Y., Zhao Y., Lin J.-F., Zhao R.-Y., Ye Z.-W., Guo L.Q. (2021). Transcriptomic Analysis of *Cordyceps militaris* and Mining of Genes Involved in Carotenoid Biosynthesis. Food Sci..

[108] Chen B.X., Wei T., Xue L.N., Zheng Q.W., Ye Z.W., Zou Y., Yang Y., Yun F., Guo L.Q., Lin J.F. (2020). Transcriptome Analysis Reveals the Flexibility of Cordycepin Network in *Cordyceps militaris* Activated by L-Alanine Addition. Front. Microbiol..

[109] Raethong N., Laoteng K., Vongsangnak W. (2018). Uncovering global metabolic response to cordycepin production in *Cordyceps militaris* through transcriptome and genome-scale network-driven analysis. Sci. Rep..

[110] Wongsa B., Raethong N., Chumnanpuen P., Wong-Ekkabut J., Laoteng K., Vongsangnak W. (2020). Alternative metabolic routes in channeling xylose to cordycepin production of *Cordyceps militaris* identified by comparative transcriptome analysis. Genomics.

[111] Yu Y.-H., Wu T.-H., Ye Z.-W., Chen B.-X., Guo L.-Q., Lin J.-F. (2020). The regulation network of cold-induced primordium formation in *Flammulina filiformis* based on transcriptome. Mycosystema.

[112] Liu X.-B., Xia E.-H., Li M., Cui Y.-Y., Wang P.-M., Zhang J.-X., Xie B.-G., Xu J.-P., Yan J.-J., Li J. (2020). Transcriptome data reveal conserved patterns of fruiting body development and response to heat stress in the mushroom-forming fungus *Flammulina filiformis*. PLoS ONE.

[113] Wang W., Chou T.-S., Liu F., Yan J.-J., Wu T.-J., Li S.-J., Xie B.-G. (2015). Comparison of gene expression patterns between the monokaryotic and dikaryotic mycelia of *Flammulina velutipes*. Mycosystema.

[114] Liu F., Wang W., Xie B.-G. (2014). Comparison of Gene Expression Patterns in the Mycelium and Primordia of *Flammulina velutipes*, Strain 1123. Acta Edulis Fungi.

[115] Cai M., Liang X., Liu Y., Hu H., Xie Y., Chen S., Gao X., Li X., Xiao C., Chen D. (2021). Transcriptional Dynamics of Genes Purportedly Involved in the Control of Meiosis, Carbohydrate, and Secondary Metabolism during Sporulation in *Ganoderma lucidum*. Genes.

[116] Ma Z., Xu M., Wang Q., Wang F., Zheng H., Gu Z., Li Y., Shi G., Ding Z. (2019). Development of an Efficient Strategy to Improve Extracellular Polysaccharide Production of *Ganoderma lucidum* Using L-Phenylalanine as an Enhancer. Front. Microbiol..

[117] You B.-J., Tien N., Lee M.-H., Bao B.-Y., Wu Y.-S., Hu T.-C., Lee H.-Z. (2017). Induction of apoptosis and ganoderic acid biosynthesis by cAMP signaling in *Ganoderma lucidum*. Sci. Rep..

[118] Yu G.-J., Wang M., Huang J., Yin Y.-L., Chen Y.-J., Jiang S., Jin Y.-X., Lan X.-Q., Wong B.H.C., Liang Y. (2012). Deep Insight into the *Ganoderma lucidum* by Comprehensive Analysis of Its Transcriptome. PLoS ONE.

[119] Nie W.-Q., Wu T.-X., Zhong M., Lu H.-Y. (2017). Transcriptome Sequencing and Analysis of Grifola frondosa Mycelia. Food Sci..

[120] Song H.-Y., Kim D.-H., Kim J.-M. (2018). Comparative transcriptome analysis of dikaryotic mycelia and mature fruiting bodies in the edible mushroom *Lentinula edodes*. Sci. Rep..

[121] Kim J.Y., Kim D.Y., Park Y.-J., Jang M.-J. (2020). Transcriptome analysis of the edible mushroom *Lentinula edodes* in response to blue light. PLoS ONE.

[122] Lei X.-E., Qiao Y.-N., Mao J.-X., Yang Z.-S., Zhang L.-L., Li L.-Y. (2020). Comparative analyses of gene expression based on transcriptome data in different laccase activities of monokaryotic mycelia of *Lentinula edodes*. Mycosystema.

[123] Huang X., Zhang R., Qiu Y., Wu H., Xiang Q., Yu X., Zhao K., Zhang X., Chen Q., Penttinen P. (2020). RNA-seq Profiling Showed Divergent Carbohydrate-Active Enzymes (CAZymes) Expression Patterns in *Lentinula edodes* at Brown Film Formation Stage Under Blue Light Induction. Front. Microbiol..

[124] Yoo S.-i., Lee H.-Y., Markkandan K., Moon S., Ahn Y.J., Ji S., Ko J., Kim S.-J., Ryu H., Hong C.P. (2019). Comparative transcriptome analysis identified candidate genes involved in mycelium browning in *Lentinula edodes*. BMC Genom..

[125] Ke S., Ding L., Niu X., Shan H., Song L., Xi Y., Feng J., Wei S., Liang Q. (2023). Comparative transcriptome analysis on candidate genes associated with fruiting body growth and development in Lyophyllum decastes. PeerJ.

[126] Fan T., Ren R., Tang S., Zhou Y., Cai M., Zhao W., He Y., Xu J. (2023). Transcriptomics combined with metabolomics unveiled the key genes and metabolites of mycelium growth in Morchella importuna. Front. Microbiol..

[127] Hao H., Zhang J., Wang H., Wang Q., Chen M., Juan J., Feng Z., Chen H. (2019). Comparative transcriptome analysis reveals potential fruiting body formation mechanisms in Morchella importuna. AMB Express.

[128] Liu W., Cai Y., He P., Chen L., Bian Y. (2019). Comparative transcriptomics reveals potential genes involved in the vegetative growth of Morchella importuna. 3 Biotech.

[129] Wang J.-Q., Ye H.-L., Liang D.-W., Liu D.-Y., Geng F., Li X. (2018). De Novo Sequencing and Transcriptome Analysis of Morchella importuna Fruiting Body. Food Sci..

[130] Zhao Y., Zhang J., Meng Q., Zhang H., Zhou G., Li M., Wu P., Shu R., Gao X., Guo L. (2020). Transcriptomic analysis of the orchestrated molecular mechanisms underlying fruiting body initiation in Chinese cordyceps. Gene.

[131] Huang Y.-Q., Tong X.-X., Tao X., Wang Y.-X., Peng C., Wang S.-H., Guo J.-L. (2017). Study on bio-synthesis of cordycepin in *Ophiocordyceps sinensis* based on RNA-seq. Chin. Tradit. Herb. Drugs.

[132] Li F., Chen L.-D., Ai L.-Y., Liu Y.-C., Yan M., Sun S.-J. (2018). Comparative transcriptomics analyses of *Pleurotus eryngii* at different developmental stages. Mycosystema.

[133] Cao J., Sun M., Yu M., Xu Y., Xie J., Zhang H., Chen J., Xu T., Qian X., Sun S. (2023). Transcriptome Analysis Reveals the Function of a G-Protein &alpha; Subunit Gene in the Growth and Development of *Pleurotus eryngii*. J. Fungi.

[134] Xie C., Gong W., Zhu Z., Yan L., Hu Z., Peng Y. (2018). Comparative transcriptomics of *Pleurotus eryngii* reveals blue-light regulation of carbohydrate-active enzymes (CAZymes) expression at primordium differentiated into fruiting body stage. Genomics.

[135] Fu Y.-P., Liang Y., Dai Y.-T., Yang C.-T., Duan M.-Z., Zhang Z., Hu S.-N., Zhang Z.-W., Li Y. (2016). De Novo Sequencing and Transcriptome Analysis of *Pleurotus eryngii* subsp. *tuoliensis* (Bailinggu) Mycelia in Response to Cold Stimulation. Molecules.

[136] Hou L., Yan K., Dong S., Guo L., Liu J., Wang S., Chang M., Meng J. (2023). Transcriptome Analysis Revealed That Hydrogen Peroxide-Regulated Oxidative Phosphorylation Plays an Important Role in the Formation of *Pleurotus ostreatus* Cap Color. J. Fungi.

[137] Wang L., Gao W., Wu X., Zhao M., Qu J., Huang C., Zhang J. (2018). Genome-Wide Characterization and Expression Analyses of *Pleurotus ostreatus* MYB Transcription Factors during Developmental Stages and under Heat Stress Based on de novo Sequenced Genome. Int. J. Mol. Sci..

[138] Wang W.-K., Song J.-L., Lu N., Yuan W.-D., Yan J., Chen G.-P. (2020). Cloning and expression of thePpFBD1 involved in primordium formation ofPleurotus pulmonarius. Acta Agric. Zhejiangensis.

[139] Fu Y., Dai Y., Yang C., Wei P., Song B., Yang Y., Sun L., Zhang Z.-W., Li Y. (2017). Comparative Transcriptome Analysis Identified Candidate Genes Related to Bailinggu Mushroom Formation and Genetic Markers for Genetic Analyses and Breeding. Sci. Rep..

[140] Du F., Ya Li mai mai ti N.E.z.y., Hu Q., Zou Y., Ye D., Zhang H. (2019). A Comparative Transcriptome Analysis Reveals Physiological Maturation Properties of Mycelia in Pleurotus tuoliensis. Genes.

[141] Rong C.-B., Zhao S., Song S., Wang J., Liu Y. (2018). Identification of Candidate Genes Related to the Development of Pleurotus tuoliensis Fruiting Bodies. Biotechnol. Bull..

[142] Yang C., Ma L., Xiao D., Ying Z., Jiang X., Lin Y. (2020). Integration of ATAC-Seq and RNA-Seq Identifies Key Genes in Light-Induced Primordia Formation of Sparassis latifolia. Int. J. Mol. Sci..

[143] Xiao D.-L., Zhang D., Ma L., Wang H.-Y., Lin Y.-S. (2017). Preliminary Study on Differentially Expressed Genes of Sparassis latifolia under Light Inducing. Edible Fungi China.

[144] Hao H., Zhang J., Wu S., Bai J., Zhuo X., Zhang J., Kuai B., Chen H. (2022). Transcriptomic analysis of *Stropharia rugosoannulata* reveals carbohydrate metabolism and cold resistance mechanisms under low-temperature stress. AMB Express.

[145] Wang D., Niu B., Song J., Zhao S.-H., Lei S.-R., Guo L.-A., Zhang F.-L., Liu W.-J., Chang L.-J., Zhao L.-M. (2019). Study on Fructose and Mannose Metabolism Pathway ofTremella fuciformisBased on Transcriptome. Southwest China J. Agric. Sci..

[146] Liu M., Yu T., Singh P.K., Liu Q., Liu H., Zhu Q., Xiao Z., Xu J., Peng Y., Fu S. (2020). A Comparative Transcriptome Analysis of *Volvariella volvacea* Identified the Candidate Genes Involved in Fast Growth at the Mycelial Growth Stage. Genes.

[147] Shu S., Chen B., Zhou M., Zhao X., Xia H., Wang M. (2013). De Novo Sequencing and Transcriptome Analysis of Wolfiporia cocos to Reveal Genes Related to Biosynthesis of Triterpenoids. PLoS ONE.

[148] Li J., Wu B., Xu J., Liu C. (2014). Genome-Wide Identification and Characterization of Long Intergenic Non-Coding RNAs in *Ganoderma lucidum*. PLoS ONE.

[149] Qu J., Zhao M., Hsiang T., Feng X., Zhang J., Huang C. (2016). Identification and Characterization of Small Noncoding RNAs in Genome Sequences of the Edible Fungus *Pleurotus ostreatus*. Biomed Res. Int..

[150] Zhou Y., Fan X.-Z., Chen L.-F., Bian Y.-B. (2014). Distribution and sequence characteristics of SSR in the transcriptomes of Auricularia auricula-judae and Auricularia polytricha. Mycosystema.

[151] Ma J., Qu W., Chen C.-Y., Wang L., Ma W., Liu Z.-S., Ma J., Yang S., Ding L., Gao Q. (2020). Development of EST-SSR markers based on transcriptome sequencing ofMorchellaspp. and its genetic diversity analysis. Jiangsu J. Agric. Sci..

[152] Zhang Y., Mo M., Yang L., Mi F., Cao Y., Liu C., Tang X., Wang P., Xu J. (2021). Exploring the Species Diversity of Edible Mushrooms in Yunnan, Southwestern China, by DNA Barcoding. J. Fungi.

[153] Yu F.-M., Jayawardena R.S., Thongklang N., Lv M.-L., Zhu X.-T., Zhao Q. (2022). Morel Production Associated with Soil Nitrogen-Fixing and Nitrifying Microorganisms. J. Fungi.

[154] Park Y.-J., Baek J.H., Lee S., Kim C., Rhee H., Kim H., Seo J.-S., Park H.-R., Yoon D.-E., Nam J.-Y. (2014). Whole Genome and Global Gene Expression Analyses of the Model Mushroom *Flammulina velutipes* Reveal a High Capacity for Lignocellulose Degradation. PLoS ONE.

[155] Park M., Kim M., Kim S., Ha B., Ro H.-S. (2015). Differential Expression of Laccase Genes in *Pleurotus ostreatus* and Biochemical Characterization of Laccase Isozymes Produced in Pichia pastoris. Mycobiology.

[156] Tang X., Ding X., Hou Y.-l. (2020). Comparative analysis of transcriptomes revealed the molecular mechanism of development of Tricholoma matsutake at different stages of fruiting bodies. Food Sci. Biotechnol..

[157] Wang M., Gu B., Huang J., Jiang S., Chen Y., Yin Y., Pan Y., Yu G., Li Y., Wong B.H.C. (2013). Transcriptome and Proteome Exploration to Provide a Resource for the Study of Agrocybe aegerita. PLoS ONE.

[158] Li J., Li Z., Zhao T., Yan X., Pang Q. (2016). Proteomic Analysis of Auricularia auricula-judae Under Freezing Treatment Revealed Proteins and Pathways Associated With Melanin Reduction. Front. Microbiol..

[159] Xu L., Lin R., Li X., Zhang C., Yang X., Guo L., Yu H., Gao X., Hu C. (2023). Comparative Proteomic Analyses within Three Developmental Stages of the Mushroom White Hypsizygus marmoreus. J. Fungi.

[160] Yang X., Lin R., Xu K., Guo L., Yu H. (2021). Comparative Proteomic Analysis within the Developmental Stages of the Mushroom White Hypsizygus marmoreus. J. Fungi.

[161] Xu L., Guo L., Yu H. (2021). Label-Free Comparative Proteomics Analysis Revealed Heat Stress Responsive Mechanism in Hypsizygus marmoreus. Front. Microbiol..

[162] Zhao Y., Li H., Yao Y., Wei Q., Hu T., Li X., Zhu B., Ma H. (2025). Combined analysis of proteomics and metabolism reveals critical roles of oxidoreductase activity in mushrooms stimulated by wolfberry and sea buckthorn substrates. Front. Nutr..

[163] Cai Y., Liu W., Zhang Q., Lu D., Cai D., Zhao Y., Ma X. (2022). Label-free based comparative proteomic analysis of Morchella importuna development from the vegetative to the sexual reproductive stages. J. Agric. Food Res..

[164] Tong X., Wang F., Zhang H., Bai J., Dong Q., Yue P., Jiang X., Li X., Wang L., Guo J. (2021). iTRAQ-based comparative proteome analyses of different growth stages revealing the regulatory role of reactive oxygen species in the fruiting body development of *Ophiocordyceps sinensis*. PeerJ.

[165] Desiderio A., Goppa L., Santambrogio C., Brocca S., Buratti S., Girometta C.E., Sarkar M., Venuti M.T., Savino E., Rossi P. (2025). Improving the Proteome-Mining of Schizophyllum commune to Enhance Medicinal Mushroom Applications. J. Fungi.

[166] Li Y., Tang H., Zhao W., Yang Y., Fan X., Zhan G., Li J., Sun S. (2022). Study of Dimorphism Transition Mechanism of Tremella fuciformis Based on Comparative Proteomics. J. Fungi.

[167] Zha L., Chen M., Guo Q., Tong Z., Li Z., Yu C., Yang H., Zhao Y. (2022). Comparative Proteomics Study on the Postharvest Senescence of *Volvariella volvacea*. J. Fungi.

[168] Wang C., Zhang X., Zeng Z., Song F., Lin Z., Chen L., Cai Z. (2022). Transcriptome Analysis Explored the Differential Genes’ Expression During the Development of the *Stropharia rugosoannulata* Fruiting Body. Front. Genet..

[169] Xiong C., Xia Y., Zheng P., Shi S., Wang C. (2010). Developmental stage-specific gene expression profiling for a medicinal fungus *Cordyceps militaris*. Mycology.

[170] Gong M., Wang Y., Zhang J., Zhao Y., Wan J., Shang J., Yang R., Wu Y., Li Y., Tan Q. (2020). Chilling Stress Triggers VvAgo1-Mediated miRNA-Like RNA Biogenesis in *Volvariella volvacea*. Front. Microbiol..

[171] Liang X., Han J., Cui Y., Shu X., Lei M., Wang B., Jia D., Peng W., He X., Liu X. (2025). Whole-Genome Sequencing of *Flammulina filiformis* and Multi-Omics Analysis in Response to Low Temperature. J Fungi.

[172] Krah F.-S., Hess J., Hennicke F., Kar R., Bässler C. (2021). Transcriptional response of mushrooms to artificial sun exposure. Ecol. Evol..

[173] Wang Y., Mao C., Shi Y., Fan X., Sun L., Zhuang Y. (2022). Transcriptome analysis of the response of Hypomyces chrysospermus to cadmium stress. Front. Microbiol..

[174] Chen C., Fu R., Wang J., Li X., Chen X., Li Q., Lu D. (2021). Genome sequence and transcriptome profiles of pathogenic fungus Paecilomyces penicillatus reveal its interactions with edible fungus Morchella importuna. Comput. Struct. Biotechnol. J..

[175] Xu Z., Liu J.-Y., Zhang D., Wang R.-J., Pan Y.-J., Tan Q., Shang X.-D. (2016). Transcriptome Comparison of Differentially Expressed Genes in the Mycelial Stages of *Flammulina velutipes* Monokaryon 3_M and the Hybrid Dikaryon G1. Acta Edulis Fungi.

[176] Gehrmann T., Pelkmans J.F., Ohm R.A., Vos A.M., Sonnenberg A.S.M., Baars J.J.P., Wösten H.A.B., Reinders M.J.T., Abeel T. (2018). Nucleus-specific expression in the multinuclear mushroom-forming fungus *Agaricus bisporus* reveals different nuclear regulatory programs. Proc. Natl. Acad. Sci. USA.

[177] Liu T., Li H., Ding Y., Qi Y., Gao Y., Song A., Shen J., Qiu L. (2017). Genome-wide gene expression patterns in dikaryon of the basidiomycete fungus *Pleurotus ostreatus*. Braz. J. Microbiol..

[178] Alfaro M., Castanera R., Lavín J.L., Grigoriev I.V., Oguiza J.A., Ramírez L., Pisabarro A.G. (2016). Comparative and transcriptional analysis of the predicted secretome in the lignocellulose-degrading basidiomycete fungus *Pleurotus ostreatus*. Environ. Microbiol..

[179] Tang L.-H., Bao D.-P., Wan J.-N., Li Y., Wang Y., Tan Q. (2016). Transcriptome analysis of transcription factors activiated at the light-induced brown mycelial film formation stage in *Lentinula edodes*. Mycosystema.

[180] Fang D., Zheng Z., Ma N., Yang W., Dai C., Zhao M., Deng Z., Hu Q., Zhao L. (2020). Label-free proteomic quantification of packaged *Flammulina filiformis* during commercial storage. Postharvest Biol. Technol..

[181] Ijoma G.N., Heri S.M., Matambo T.S., Tekere M. (2021). Trends and Applications of Omics Technologies to Functional Characterisation of Enzymes and Protein Metabolites Produced by Fungi. J. Fungi.

[182] Yang H., Zheng Z., Zhou H., Qu H., Gao H. (2022). Proteomics Reveals the Mechanism Underlying the Autolysis of Postharvest Coprinus comatus Fruiting Bodies. J. Agric. Food Chem..

[183] Lin R., Zhang L., Yang X., Li Q., Zhang C., Guo L., Yu H., Yu H. (2022). Responses of the Mushroom *Pleurotus ostreatus* under Different CO_2_ Concentration by Comparative Proteomic Analyses. J. Fungi.

[184] Yu H., Jiang N., Yan M., Cheng X., Zhang L., Zhai D., Liu J., Zhang M., Song C., Yu H. (2023). Comparative analysis of proteomes and transcriptomes revealed the molecular mechanism of development and nutrition of *Pleurotus giganteus* at different fruiting body development stages. Front. Nutr..

[185] Vita F., Giuntoli B., Bertolini E., Taiti C., Marone E., D’Ambrosio C., Trovato E., Sciarrone D., Zoccali M., Balestrini R. (2020). Tuberomics: A molecular profiling for the adaption of edible fungi (*Tuber magnatum Pico*) to different natural environments. BMC Genom..

[186] Zhao Y., Yao Y., Li H., Han Z., Ma X. (2024). Integrated transcriptome and metabolism unravel critical roles of carbon metabolism and oxidoreductase in mushroom with Korshinsk peashrub substrates. BMC Genom..

[187] Horie K., Rakwal R., Hirano M., Shibato J., Nam H.W., Kim Y.S., Kouzuma Y., Agrawal G.K., Masuo Y., Yonekura M. (2008). Proteomics of two cultivated mushrooms Sparassis crispa and Hericium erinaceum provides insight into their numerous functional protein components and diversity. J. Proteome Res..

[188] Wu M.-X., Zou Y., Yu Y.-H., Chen B.-X., Zheng Q.-W., Ye Z.-W., Wei T., Ye S.-Q., Guo L.-Q., Lin J.-F. (2021). Comparative transcriptome and proteome provide new insights into the regulatory mechanisms of the postharvest deterioration of Pleurotus tuoliensis fruitbodies during storage. Food Res. Int..

[189] Wang S., Wang J., Wang T., Li T., Xu L., Cheng Y., Chang M., Meng J., Hou L. (2024). Integrated Transcriptomics–Proteomics Analysis Reveals the Response Mechanism of Morchella sextelata to Pseudodiploöspora longispora Infection. J. Fungi.

[190] Ma X., Lu L., Yao F., Fang M., Wang P., Meng J., Shao K., Sun X., Zhang Y. (2023). High-quality genome assembly and multi-omics analysis of pigment synthesis pathway in Auricularia cornea. Front. Microbiol..

[191] Lu Y., Luo F., Cen K., Xiao G., Yin Y., Li C., Li Z., Zhan S., Zhang H., Wang C. (2017). Omics data reveal the unusual asexual-fruiting nature and secondary metabolic potentials of the medicinal fungus Cordyceps cicadae. BMC Genom..

[192] Chen Y., Wu Y., Liu L., Feng J., Zhang T., Qin S., Zhao X., Wang C., Li D., Han W. (2019). Study of the whole genome, methylome and transcriptome of *Cordyceps militaris*. Sci. Rep..

[193] Yin Y., Yu G., Chen Y., Jiang S., Wang M., Jin Y., Lan X., Liang Y., Sun H. (2012). Genome-wide transcriptome and proteome analysis on different developmental stages of *Cordyceps militaris*. PLoS ONE.

[194] Chen J., Li J.-M., Tang Y.-J., Ma K., Li B., Zeng X., Liu X.-B., Li Y., Yang Z.-L., Xu W.-N. (2020). Genome-wide analysis and prediction of genes involved in the biosynthesis of polysaccharides and bioactive secondary metabolites in high-temperature-tolerant wild *Flammulina filiformis*. BMC Genom..

[195] Wang R.-Q., Yan J.-J., Li Y.-N., Yang H., Ma X.-B., Wang M., Tao Y.-X., Xie B.-G. (2020). Cytochrome c peroxidase gene(ffccp) and its differential expression during stipe elongation in *Flammulina filiformis*. Mycosystema.

[196] Gao Y., Wang Y., Qian J., Si W., Tan Q., Xu J., Zhao Y. (2020). Melatonin enhances the cadmium tolerance of mushrooms through antioxidant-related metabolites and enzymes. Food Chem..

[197] Wang R., Ma P., Li C., Xiao L., Liang Z., Dong J. (2019). Combining transcriptomics and metabolomics to reveal the underlying molecular mechanism of ergosterol biosynthesis during the fruiting process of *Flammulina velutipes*. BMC Genom..

[198] Zhu Y., Luo H., Zhang X., Song J., Sun C., Ji A., Xu J., Chen S. (2014). Abundant and Selective RNA-Editing Events in the Medicinal Mushroom *Ganoderma lucidum*. Genetics.

[199] Zhou S., Zhang J., Ma F., Tang C., Tang Q., Zhang X. (2018). Investigation of lignocellulolytic enzymes during different growth phases of *Ganoderma lucidum* strain G0119 using genomic, transcriptomic and secretomic analyses. PLoS ONE.

[200] Zhang J., Li Y., Mao Y., Zhang Y., Zhou B., Liu W., Wang W., Zhang C. (2025). Integrated Transcriptomic and Proteomic Analyses Reveal Molecular Mechanism of Response to Heat Shock in Morchella sextelata. J. Fungi.

[201] Wen J., Zhang Z., Gong L., Xun H., Li J., Qi B., Wang Q., Li X., Li Y., Liu B. (2019). Transcriptome Changes during Major Developmental Transitions Accompanied with Little Alteration of DNA Methylome in Two Pleurotus Species. Genes.

[202] Wan J.N., Li Y., Guo T., Ji G.Y., Luo S.Z., Ji K.P., Cao Y., Tan Q., Bao D.P., Yang R.H. (2021). Whole-Genome and Transcriptome Sequencing of Phlebopus portentosus Reveals Its Associated Ectomycorrhizal Niche and Conserved Pathways Involved in Fruiting Body Development. Front. Microbiol..

[203] Fernández-Fueyo E., Ruiz-Dueñas F.J., López-Lucendo M.F., Pérez-Boada M., Rencoret J., Gutiérrez A., Pisabarro A.G., Ramírez L., Martínez A.T. (2016). A secretomic view of woody and nonwoody lignocellulose degradation by *Pleurotus ostreatus*. Biotechnol. Biofuels.

[204] Thongbai B., Rapior S., Hyde K.D., Wittstein K., Stadler M. (2015). *Hericium erinaceus*, an amazing medicinal mushroom. Mycol. Prog..

[205] Feng W., Guo Z., Jin Q., Xu F., Shen Y., Song T., Wang M., Zhang J., Fan L., Huang X. (2025). A Preliminary Exploration of Transcriptome and Proteomic Changes During the Young and Harvest Periods in Morchella sextelata. J. Fungi.

[206] Ballard J.L., Wang Z., Li W., Shen L., Long Q. (2024). Deep learning-based approaches for multi-omics data integration and analysis. BioData Min..

[207] Cheawchanlertfa P., Chitcharoen S., Raethong N., Liu Q., Chumnanpuen P., Soommat P., Song Y., Koffas M., Laoteng K., Vongsangnak W. (2022). Enhancing Genome-Scale Model by Integrative Exometabolome and Transcriptome: Unveiling Carbon Assimilation towards Sphingolipid Biosynthetic Capability of *Cordyceps militaris*. J. Fungi.

[208] Soommat P., Raethong N., Ruengsang R., Thananusak R., Laomettachit T., Laoteng K., Saithong T., Vongsangnak W. (2024). Light-Exposed Metabolic Responses of *Cordyceps militaris* through Transcriptome-Integrated Genome-Scale Modeling. Biology.

[209] Wang F., Li F., Han L., Wang J., Ding X., Liu Q., Jiang M., Li H. (2024). High-Yield-Related Genes Participate in Mushroom Production. J. Fungi.

[210] Sakamoto Y., Sato S., Takizawa M., Goto F., Eda K., Yamauchi T. (2022). Marker-assisted selection of a novel *Lentinula edodes* cultivation strain with improved post-harvest quality. Mushroom Sci. Biotechnol..

[211] Li W., Zou G., Bao D., Wu Y. (2024). Current Advances in the Functional Genes of Edible and Medicinal Fungi: Research Techniques, Functional Analysis, and Prospects. J. Fungi.

[212] Yang Y., Saand M.A., Huang L., Abdelaal W.B., Zhang J., Wu Y., Li J., Sirohi M.H., Wang F. (2021). Applications of Multi-Omics Technologies for Crop Improvement. Front. Plant Sci..

[213] Zhang Y., Tian L., Xu M.-H., Wang B., Song B., Li Y. (2020). Research progress in comprehensive utilization of spent mushroom substrates. Microbiol. China.

[214] Zeb U., Aziz T., Azizullah A., Zan X.Y., Khan A.A., Bacha S.A.S., Cui F.J. (2024). Complete mitochondrial genomes of edible mushrooms: Features, evolution, and phylogeny. Physiol. Plant..

